# From immunological mechanisms to targeted therapies: a bibliometric analysis of vitiligo treatment research (2005–2025)

**DOI:** 10.3389/fimmu.2025.1733857

**Published:** 2026-01-14

**Authors:** Longfei Luo, Xiaolei He, Ying Shi

**Affiliations:** Department of Dermatology, Renmin Hospital of Wuhan University, Wuhan, China

**Keywords:** autoimmune disease, bibliometric analysis, CD8+ T cells, IFN-γ signaling, immunotherapy, JAK-STAT pathway, translational immunology, vitiligo

## Abstract

**Background:**

Vitiligo is a chronic autoimmune disorder characterized by CD8+ T cell-mediated destruction of melanocytes. It affects up to 2% of the global population and has a profound psychosocial impact. Over the past two decades, advances in understanding immunological mechanisms, particularly IFN-γ signaling and JAK-STAT pathway dysregulation, have reshaped therapeutic approaches from empirical regimens to targeted immunomodulatory interventions.

**Objective:**

To analyze global vitiligo treatment research trends from 2005 to 2025, examining publication patterns, collaboration networks, and the evolution of therapeutic strategies.

**Methods:**

Publications related to vitiligo treatment were retrieved from the Web of Science Core Collection (WoSCC) and analyzed using R (Bibliometrix), VOSviewer, and CiteSpace. Scopus database was used for supplementary validation of major trends. In parallel, randomized controlled trials (RCTs) were systematically identified from PubMed to contextualize clinical research.

**Results:**

In total, 3, 483 publications were included in this study. Annual output increased at a compound growth rate of 7.27%. China, the United States, and India were the leading contributors, whereas the Egyptian Knowledge Bank and Harvard University were the most productive institutions. Thematic analysis revealed a gradual transition from traditional therapies such as corticosteroids and phototherapy to immune-targeted strategies. Citation bursts highlight the emergence of JAK inhibitors and biologics. Network analyses revealed fragmented author clusters, but strong international collaborations, particularly in Europe. An analysis of 249 RCTs revealed that pharmacological therapies, surgical interventions, and phototherapy constitute the three major research themes. Immunomodulatory therapies, particularly JAK inhibitors, such as ritlecitinib, have emerged as a key focus in ongoing clinical trials.

**Conclusion:**

This multi-database analysis delineates the evolving landscape of vitiligo treatment research, characterized by expanding output and increasing focus on immune-targeted therapies. Persistent gaps in clinical evidence, especially regarding long-term outcomes and pediatric populations, underscore the need for continued high-quality clinical studies in this field.

## 1 Introduction

Vitiligo is a chronic autoimmune skin disorder characterized by progressive melanocyte destruction mediated by autoreactive CD8+ T cells, resulting in depigmented patches that affect 0.5–2% of the global population (1). The disease causes significant psychosocial distress, impairing quality of life and social functioning (2). Traditional treatments, including corticosteroids and narrowband UV-B phototherapy, provide partial benefits but are often unsatisfactory due to relapse and variable responses (3, 4).

Over the last decade, remarkable progress has been made in understanding vitiligo immunopathogenesis. Central to disease development are autoreactive CD8^+^ T cells, interferon-γ (IFN-γ) signaling, and the JAK-STAT pathway (5). These immunological insights have paved the way for the development of novel targeted immunotherapies. In 2022, the FDA approved topical ruxolitinib, a JAK1/2 inhibitor, as the first therapy specifically indicated for nonsegmental vitiligo (6). This milestone exemplifies the successful translation from bench discoveries to bedside applications.

Concurrently, vitiligo research has extended to adjacent fields of immunology. Reports of vitiligo-like depigmentation in patients with cancer treated with immune checkpoint inhibitors have reinforced the role of T-cell–mediated autoimmunity and offer opportunities for cross-disciplinary therapeutic exploration (7). Such developments underscore the need to map global research activities to identify emerging immunotherapeutic trends and to guide future drug development.

Bibliometric analysis offers a systematic method for evaluating publication patterns, collaborations, and research hotspots (8). Prior bibliometric studies on vitiligo have mainly focused on epidemiology and general research output (9, 10). Few studies have addressed treatment-specific trends, particularly in the evolution of immunological and molecular therapeutics. Therefore, this study provides a comprehensive bibliometric analysis of vitiligo treatment research from 2005 to 2025, with a special focus on translational immunology.

## 2 Materials and methods

### 2.1 Data source and search strategy

We employed a tiered multi-database strategy rather than full database merging due to methodological considerations. The Web of Science Core Collection (WoSCC) was selected as the primary database for comprehensive bibliometric analysis because of its standardized citation indexing and compatibility with established bibliometric tools (Bibliometrix, CiteSpace, VOSviewer). Scopus was used for cross-validation of major trends (publication growth, geographic distribution, and leading institutions) to ensure the robustness of findings across databases with different coverage profiles. PubMed was queried separately using Medical Subject Headings terms and publication type filters to specifically identify randomized controlled trials (RCTs), as its biomedical focus and standardized indexing make it optimal for retrieving high-quality clinical trials (11). This tiered approach balances the comprehensive analysis depth with a cross-database validation breadth.

The Web of Science (WoS) platform encompasses valuable sources of research papers and citations across numerous disciplines. Because of its origin in citation indexing, it helps with bibliometric analyses (12). Therefore, the literature of this study was collected from the Web of Science Core Collection (WoSCC) on August 20, 2025, covering the period from January 1, 2005, to August 20, 2025, with the following strategy: TS1=“Vitiligo*” OR “Vitiligo disease*” OR “Leukoderma*” OR “Skin Depigmentation”; TS2=“Treatment*” OR “Therapy*” OR “Management*” OR “Intervention” OR “Cure*” OR “Relief” OR “Pharmacological Treatment” OR “Topical Treatment” OR “Systemic Treatment” OR “Phototherapy” OR “Laser Treatment” OR “Immunotherapy” OR “Biologics” OR “Corticosteroid Treatment” OR “Calcipotriol” OR “Ultraviolet Therapy” OR “Corticosteroids” OR “Immunosuppressive”; TS=TS1 AND TS2. The search was restricted to English-language publications, and document types were limited to articles and reviews during the screening stage.

To minimize coverage bias and check the stability of trends, we validated the key results against Scopus, which offers broader multidisciplinary coverage in comparative benchmarks (13). The queries were based on TITLE-ABS-KEY, with a search strategy consistent with that of the WoSCC.

We also searched PubMed for randomized controlled trials (RCTs) on vitiligo to ensure the inclusion of high-quality clinical evidence. The search strategy is as following: (“vitiligo” OR “leukoderma”) AND (“randomized controlled trial” OR “RCT” OR “randomized” OR “controlled trial”) AND (“treatment” OR “therapy” OR “intervention”) AND (2005:2025[pdat]). The search was conducted on October 21, 2025.

### 2.2 literature screening process

**Figure 1:**
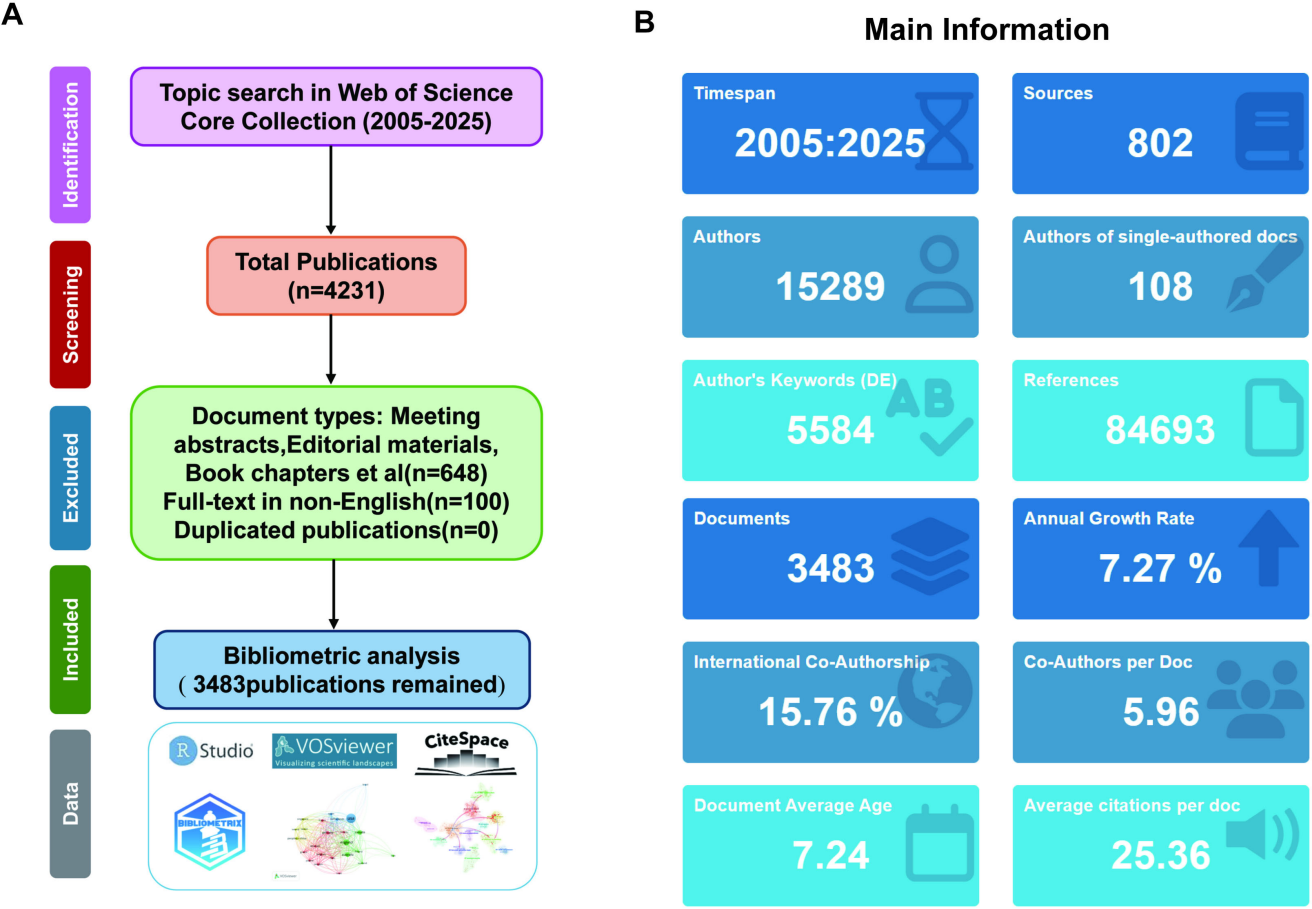
Literature screening and publication overview. **(A)** The flowchart showing the systematic screening process for vitiligo treatment publications. A total of 4, 231 records were initially identified from the Web of Science Core Collection (WoSCC) database. Following duplicate elimination, 648 non-article materials (including meeting abstracts, editorial materials, and book chapters) and 100 non-English publications were excluded, ultimately yielding 3, 483 publications eligible for bibliometric analysis. **(B)** Main information of 3, 483 publications retrieved and screened from the WoSCC database.

A systematic search identified 4, 231 publications from the Web of Science Core Collection (2005–2025). Following the Preferred Reporting Items for Systematic reviews and Meta-Analyses (PRISMA) guidelines, we applied the systematic inclusion and exclusion criteria (**Figure 1A**). We excluded 648 meeting abstracts, editorial materials, book chapters, and 100 non-English publications. By combining CiteSpace’s built-in deduplication module with manual verification by two researchers, we removed duplicates and retained 3, 483 publications for bibliometric analysis (14). All literature, including full records and cited references, was downloaded in plain text format. To minimize subjective bias, melanoma-related articles reflecting vitiligo-like adverse effects were not excluded manually (15). Similarly, a total of 6, 972 publications from Scopus were collected. A total of 491 initial records were retrieved from PubMed. Following the predefined inclusion and exclusion criteria (**Supplementary Table S1**), 259 studies were identified, and after independent screening by two authors, 249 were retained for further analysis.

### 2.3 Bibliometric analysis

For the data retrieved from WoSCC, we employed the R package bibliometrix (8) for basic statistics and collaboration analysis, CiteSpace (16) for citation burst detection and temporal analysis, and VOSviewer (17) for network visualization and clustering analysis. International collaboration (MCP) is defined as the proportion of publications with author affiliations from ≥2 countries. R bibliometrix v5.0.1 (Automatic layout was adopted to optimize node distribution, and the Walktrap algorithm was used to identify thematic clusters with high internal connectivity. The “association” mode was selected for normalization to ensure that co-occurrence strength reflects actual relevance rather than frequency bias. The top 50 high-frequency keywords were retained, with the minimum number of edges set to 2 to exclude isolated or low-correlation terms; isolated nodes were removed (Yes) to enhance overall network connectivity. The repulsion force was set to 0.1 to balance node spacing and avoid overcrowding. Node Color by Year was disabled to clearly visualize the thematic structure.), CiteSpace v6.4. R1 (time slicing=1 year; burst detection=default; pruning=pathfinder; Selection Criteria: g-index (k=25), LRF = 2.5, L/N=10, LBY = 5, e=1.0; Network: N = 624, E = 4960 (Density=0.0255)), and VOSviewer v1.6.20 (minimum keyword occurrence=5; fractional counting) (18). Microsoft Office Excel 2019 and Adobe Illustrator Artwork 16.0 were used for auxiliary analysis and drawing. Secondary mentions of vitiligo in unrelated treatment contexts were retained and flagged during qualitative synthesis to maintain analytical objectivity. For the data obtained from Scopus, we directly used its built-in “Analyze Search Results” function to perform data analysis. For RCT data retrieved from PubMed, we conducted an analysis using the Bibliometrix package in R.

## 3 Results

### 3.1 Dataset overview and publication trends

The final dataset comprised 3, 483 publications from 802 sources, including 15, 289 authors and 108 single-author publications (**Figure 1B**). The corpus contains extensive author keywords and references spanning 2005 to 2025, with substantial research impact demonstrated by citation patterns. International collaboration reached 15.76%, with nearly six coauthors per document, on average. An annual growth rate of 7.27% indicated rapid field expansion (9), and the data derived from the Scopus database exhibited a similar upward trend (**Supplementary Figure S1**).

Annual publication analysis (**Table 1**) revealed consistent publication growth, with confidence intervals (based on Polynomial Regression model) reflecting the predictive accuracy of the bibliometric model. The model-based prediction intervals illustrate the overall uncertainty range of publication growth trends, rather than serving as year-specific confidence bounds for observed counts (10).

**Table 1:** Annual publication output and model-based prediction intervals (2005–2025).

Year	Articles	Lower CI	Upper CI
2005	57	22.0877	87.4900
2006	72	36.2491	89.9747
2007	78	49.6858	94.0633
2008	77	62.2547	99.8987
2009	87	73.8868	107.5500
2010	103	84.7193	116.8800
2011	117	95.1207	127.5202
2012	127	105.5352	139.0263
2013	116	116.3231	151.0382
2014	126	127.7150	163.3250
2015	135	139.8324	175.7655
2016	175	152.7124	188.3224
2017	192	166.3179	201.0330
2018	169	180.5274	214.0185
2019	213	195.1103	227.5098
2020	231	209.7063	241.8670
2021	284	223.8712	257.5344
2022	300	237.2366	274.8805
2023	276	249.6650	294.0425
2024	316	261.2258	314.9513
2025^*^	232	272.0617	337.4640

CI, Confidence Interval; ^*^up to August 20. CI represents the 95% prediction interval derived from the fitted growth model, reflecting overall publication trend uncertainty rather than year-specific observed confidence intervals.

**Figure 2:**
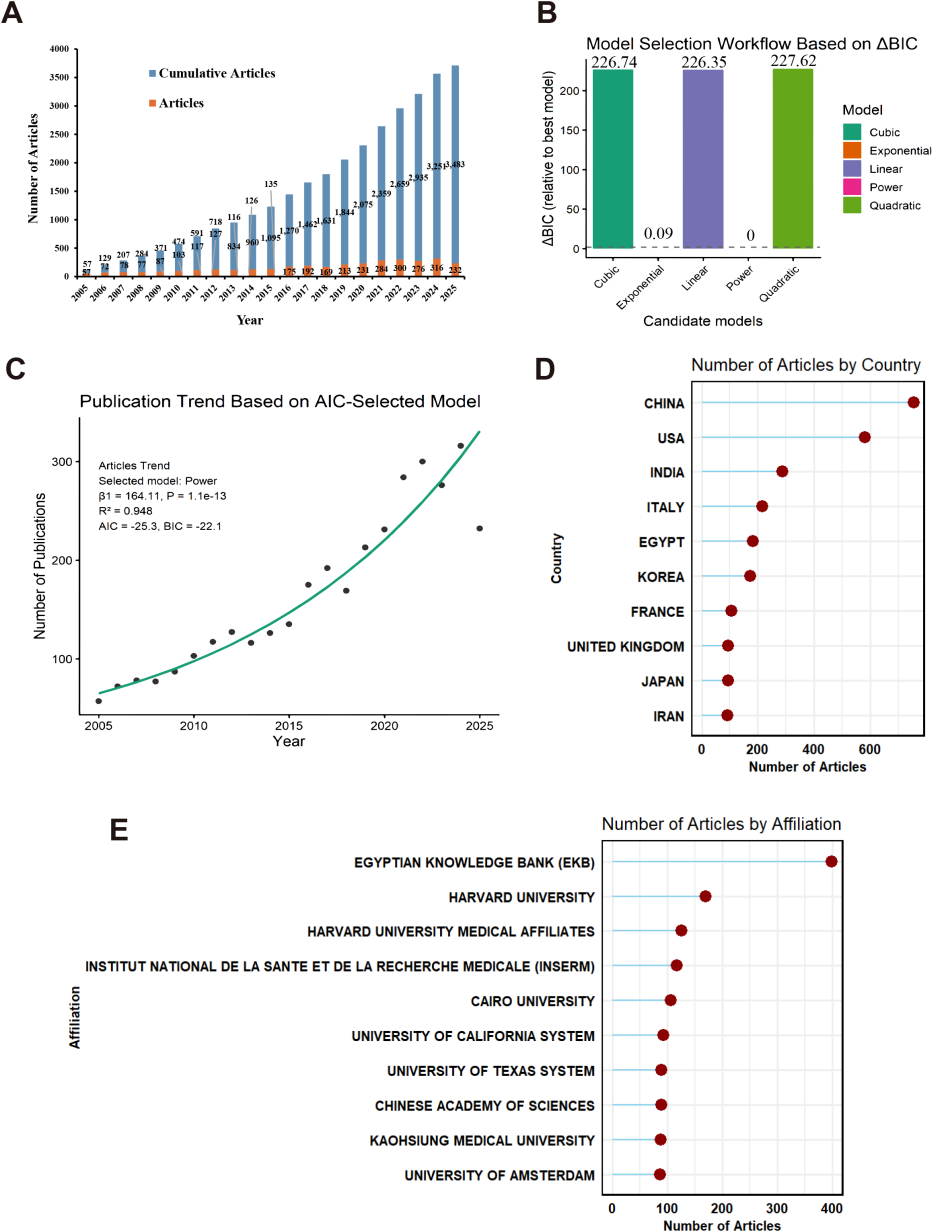
Publication trends and geographic distribution. **(A)** Annual publication trend. **(B)** Model selection workflow for publication trend fitting, evaluated by ΔBIC (difference in Bayesian Information Criterion relative to the best-fitting model). ΔBIC values (height of bars) represent the relative fit of each candidate model (Cubic, Exponential, Linear, Power, Quadratic) compared to the best model (Power, ΔBIC = 0, indicated by the dashed baseline). **(C)** Publication trend based on the AIC-selected Power model. The green curve represents the fitted trend of annual publication quantity. **(D)** Top 10 countries ranked by publication volume, with China (755 publications, 21.7%), USA (581, 16.7%), and India (287, 8.2%) as leading contributors. **(E)** Top 10 institutions by publication volume, with the Egyptian Knowledge Bank (399 publications) leading, followed by Harvard University (170) and Harvard University Medical Affiliates (125). Note the discrepancy between institutional output and research impact, as discussed in the text.

In 2005, only 57 articles were published, whereas projections for 2024 estimated over 300 publications, indicating a more than fivefold increase (**Figure 2A**).

To identify an appropriate functional form for describing publication growth, multiple candidate models were compared using the Bayesian Information Criterion (BIC) (**Figure 2B**). The power model showed the strongest support (ΔBIC = 0), while the exponential model demonstrated a nearly equivalent fit (ΔBIC = 0.09). In contrast, cubic, linear, and quadratic models exhibited substantially larger ΔBIC values (>200), indicating markedly weaker support.

Based on Akaike Information Criterion (AIC)–based selection, the power model was chosen to characterize historical publication trends (**Figure 2C**). The fitted curve closely followed observed annual publication counts and demonstrated a high goodness of fit (R² = 0.948; P = 1.1 × 10^−13^). These analyses are presented to describe historical publication dynamics and relative model performance, rather than to infer causality or to generate future projections.

### 3.2 Geographical and institutional contributions

Analysis of the national publication output revealed that China ranked first, with 755 publications, followed by the United States (581) and India (287) (**Table 2**; **Figure 2D**). The top three contributors accounted for more than 45% of global output. Other leading countries include Italy, Egypt, Korea, France, the United Kingdom, Japan, and Iran, indicating a widespread global interest in vitiligo treatment research.

**Table 2:** Top 10 countries/regions with the highest productivity and specific parameters.

Country	Articles	Articles %	SCP	MCP	MCP %
CHINA	755	21.7	700	55	7.3
USA	581	16.7	467	114	19.6
INDIA	287	8.2	269	18	6.3
ITALY	216	6.2	177	39	18.1
EGYPT	182	5.2	158	24	13.2
KOREA	172	4.9	160	12	7
FRANCE	106	3	68	38	35.8
UNITED KINGDOM	94	2.7	65	29	30.9
JAPAN	93	2.7	83	10	10.8
IRAN	92	2.6	76	16	17.4

SCP, Single Country Publications; MCP, Multiple Country Publications.

Multiple country publications (MCPs) have revealed the number of coauthors from different countries/regions (19). Although the USA had the highest MCP (n=114), its MCP ratio (MCP/articles) was only 19.6%, which was far lower than that of France (35.8%) and the United Kingdom (30.9%) (**Table 2**). Nevertheless, the USA and European countries have played a central role in shaping global research networks.

The Egyptian Knowledge Bank (EKB) led institutional output with the highest number of publications (n=399). Other top contributors included Harvard University (170), Harvard University Medical Affiliates (125), INSTITUT NATIONAL DE LA SANTE ET DE LA RECHERCHE MEDICALE (INSERM) (117), and Cairo University (106). Additional high-output institutions included the University of California (92), Chinese Academy of Sciences (89), and University of Texas (89) (**Table 3**; **Figure 2E**). The Egyptian Knowledge Bank has the highest publication volume, reflecting its role as a national aggregation platform. Among the traditional academic institutions, Harvard University has emerged as a leading contributor. Analysis based on the Scopus data also confirmed Harvard University’s leading position (**Supplementary Figure S2**). This analysis reveals the global nature of vitiligo treatment research, with contributions from institutions across multiple continents. The diversity in institutional types, from comprehensive medical centers to specialized research institutes, reflects the multidisciplinary nature of vitiligo treatment.

**Table 3:** Top 10 affiliations with the highest productivity and specific parameters.

Affiliation	Articles
EGYPTIAN KNOWLEDGE BANK (EKB)	399
HARVARD UNIVERSITY	170
HARVARD UNIVERSITY MEDICAL AFFILIATES	125
INSTITUT NATIONAL DE LA SANTE ET DE LA RECHERCHE MEDICALE (INSERM)	117
CAIRO UNIVERSITY	106
UNIVERSITY OF CALIFORNIA SYSTEM	92
CHINESE ACADEMY OF SCIENCES	89
UNIVERSITY OF TEXAS SYSTEM	89
KAOHSIUNG MEDICAL UNIVERSITY	88
UNIVERSITY OF AMSTERDAM	87

### 3.3 Interdisciplinary knowledge flow

**Figure 3:**
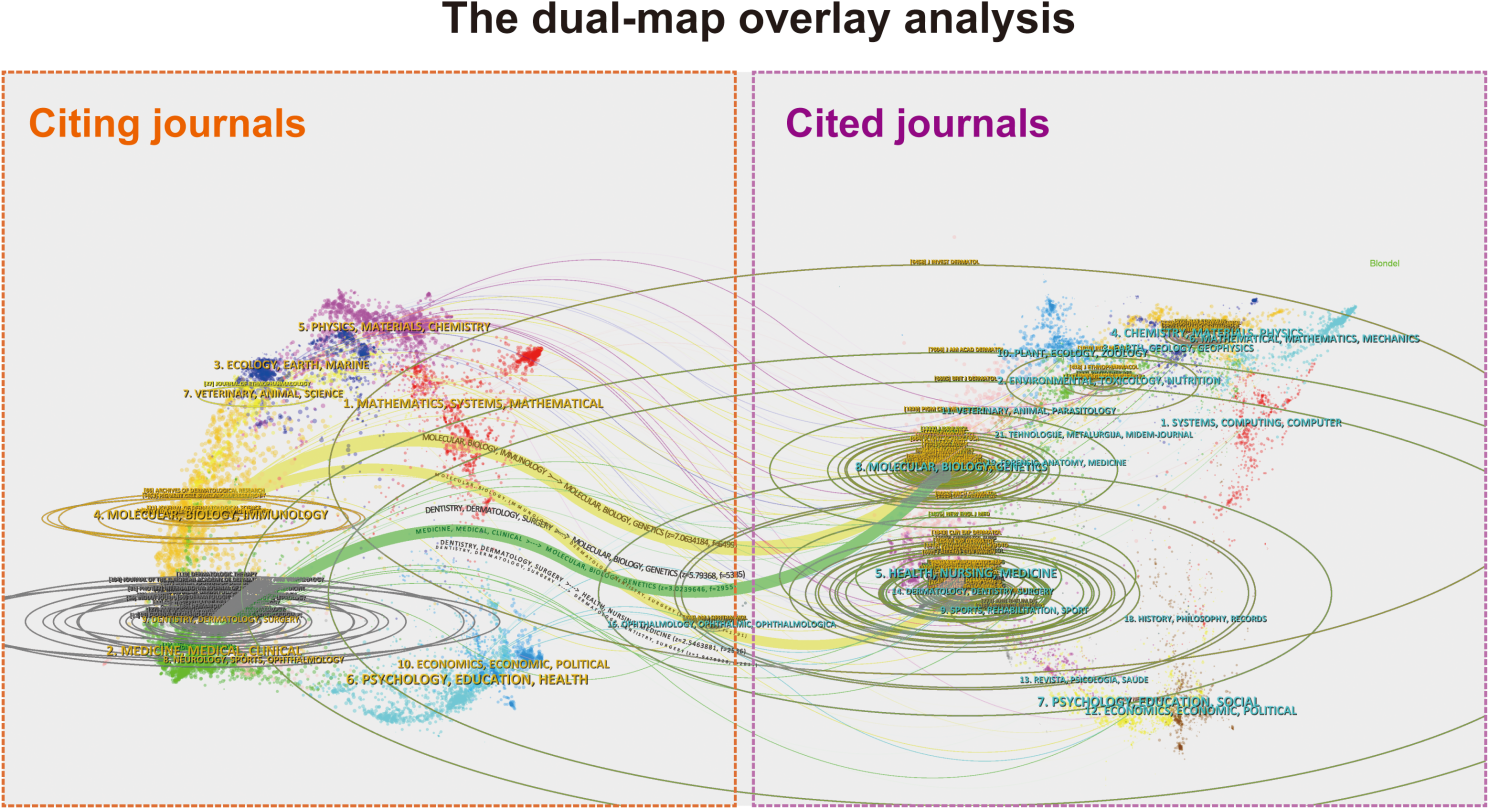
Dual-map overlay analysis of interdisciplinary knowledge flow. This visualization maps citation trajectories between citing journals (left, orange) and cited journals (right, purple), revealing the interdisciplinary nature of vitiligo research. Citation relationships are represented by the colored paths.

A dual-map overlay analysis revealed citation patterns between different journal categories (**Figure 3**). The visualization shows citing journals on the left side and cited journals on the right side, with colored citation paths indicating the knowledge flow patterns between different research fields. The colored paths trace knowledge flows between the research domains. The labels correspond to the disciplines covered by journals. The two most cited and citing fields were Molecular/Biology/Genetics, Health/Nursing/Medicine and Molecular/Biology/Immunology, Medicine/Medical/Clinical. The colored paths trace knowledge flows between the research domains. The two main yellow paths suggest that research focused on Molecular/Biology/Genetics and Dermatology/Dentistry/Surgery was frequently cited by journals in molecular/biology/immunology. Similarly, the two main green paths indicated that research from journals in molecular/biology/genetics was often cited by journals in dentistry/dermatology/surgery and medicine/medical/clinical fields. The analysis identified major citation pathways connecting clinical dermatology research with basic immunology and molecular medicine journals, reflecting the highly translational nature of vitiligo research (20).

### 3.4 Research hotspots and thematic shifts

**Figure 4:**
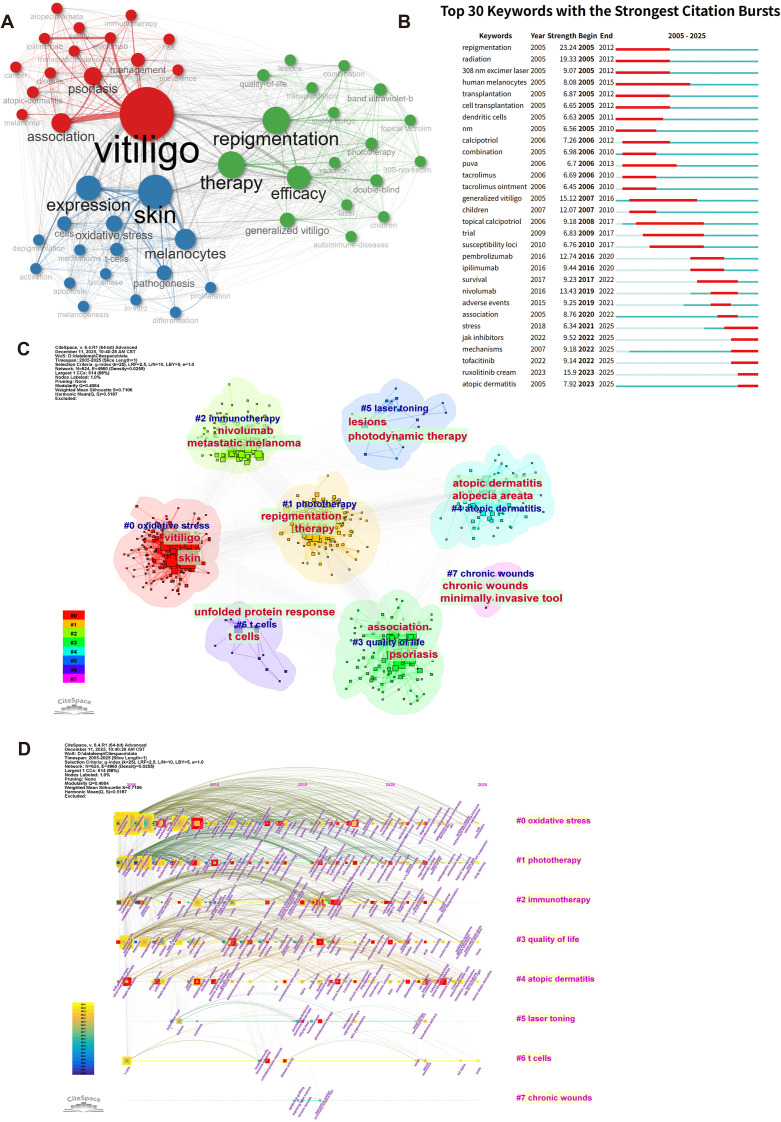
Keyword co-occurrence analysis. **(A)** Network visualization of high-frequency keywords (minimum occurrence = 5) showing clusters of related research themes. Central nodes (vitiligo, therapy, treatment, immunotherapy) represent core research areas, with peripheral nodes representing emerging topics. Node size represents keyword frequency; edge thickness represents co-occurrence strength. **(B)** Top 30 keywords with the strongest citation bursts, ranked by burst strength and duration (2005–2025). The red bars indicate citation burstiness. **(C)** Keyword clustering analysis identifying seven distinct research clusters: (#0) oxidative stress, (#1) phototherapy, (#2) immunotherapy, (#3) quality of life, (#4) atopic dermatitis, (#5) laser toning, and (#6) chronic wounds. Colors represent cluster membership; cluster labels indicate primary research focus. **(D)** Temporal keyword timeline showing the evolution of research focus from 2005 to 2025.

Co-occurrence network analysis revealed the conceptual structure of vitiligo treatment (**Figure 4A**). The network displayed “vitiligo” as the central node, with three distinct clusters emerging in different colors: red nodes focusing on clinical aspects, green nodes including “skin” and “repigmentation” terms, and blue nodes containing “therapy” and related treatment keywords. The visualization demonstrated strong connections between the central “vitiligo” node and major therapeutic concepts, reflecting the multifaceted nature of research encompassing clinical presentation, skin biology, and therapeutic interventions.

Citation burst analysis identified the top 30 keywords with the strongest citation bursts, indicating emerging research trends (**Figure 4B**). Notably, the recent emphasis on “JAK inhibitors”, “tofacitinib”, and “ruxolitinib cream” has led to a shift toward targeted immunological therapies (5).

The keyword clustering analysis identified distinct research themes, including *oxidative stress, phototherapy, immunotherapy, quality of life, atopic dermatitis, laser toning, T cells, and chronic wounds* (**Figure 4C**). Timeline analysis revealed the temporal evolution of the research keywords, revealing a clear progression from traditional therapies to targeted treatments and precision medicine approaches (**Figure 4D**). The timeline revealed three distinct research phases: traditional therapy optimization (2005–2010), mechanistic understanding development (2011–2018), and targeted therapy innovation (2019–2025) (21, 22). Notably, emerging terms such as “cellular senescence”, “Nrf2”, “regenerative medicine”, “drug delivery”, and “transdermal delivery” have appeared in recent years (**Figure 4D**), with the aim of exploring directions for future research.

### 3.5 Collaboration networks and research communities

**Figure 5:**
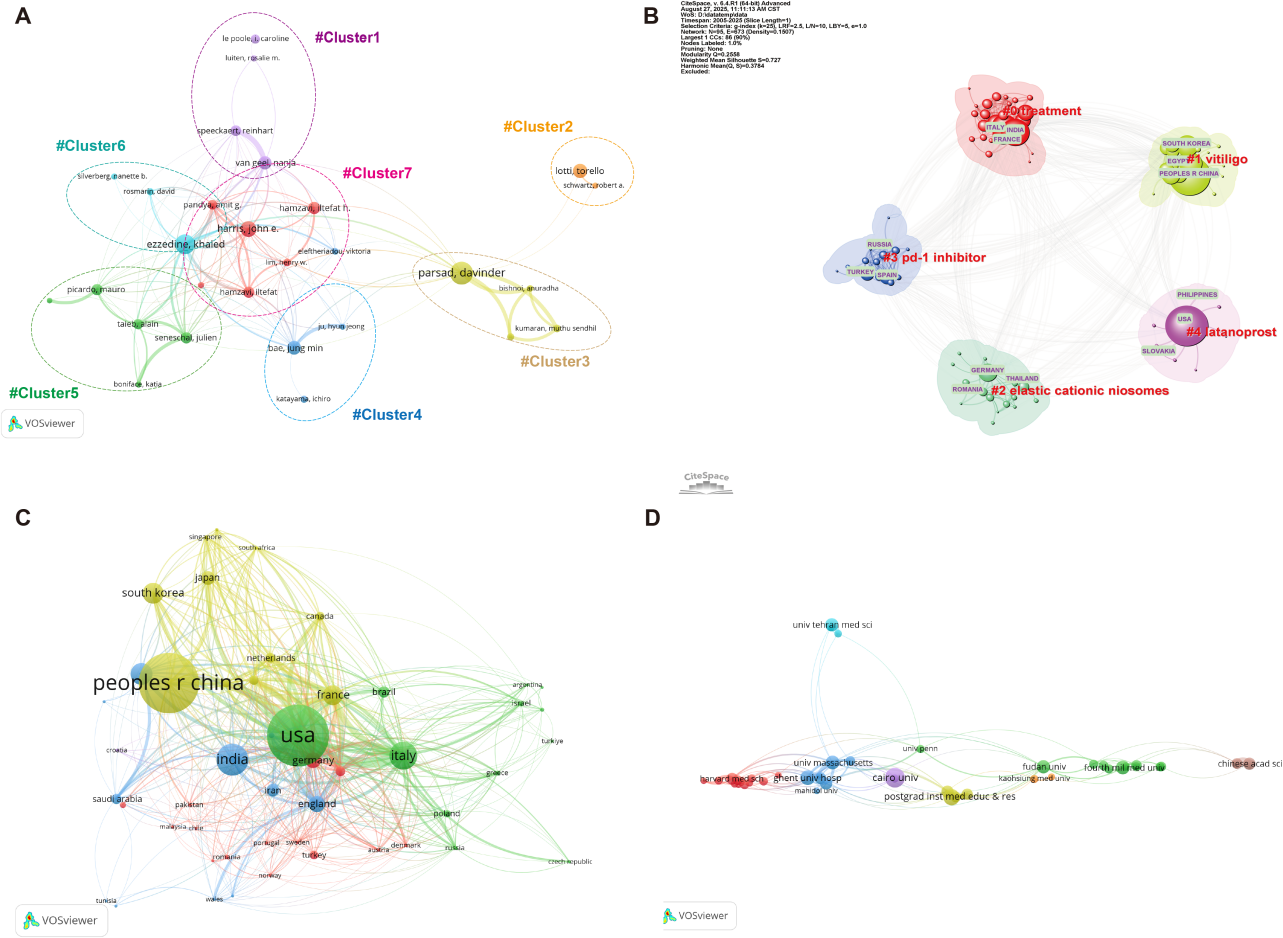
Collaboration networks in vitiligo treatment research. **(A)** Author collaboration network showing fragmented clusters with limited inter-cluster connections, indicating that vitiligo treatment research is conducted by relatively isolated research groups. Node size represents author productivity (number of publications); Line thickness indicates the strength of the connection. **(B)** Country clustering analysis revealing geographic research communities, with distinct clusters for different themes. **(C)** Country collaboration network showing international partnerships, with node size indicating national publication volume and line thickness denoting inter-country collaboration strength. **(D)** Institutional collaboration network identifying major research hubs and their collaborative relationships.

The collaborative landscape of vitiligo treatment research was analyzed from the perspectives of authorship and national and institutional cooperation (**Figure 5**). The author collaboration network (**Figure 5A**), constructed via VOSviewer and limited to the top 28 most prolific authors in vitiligo treatment research, revealed distinct co-authorship clusters. Strong intragroup linkages were observed within these clusters, indicating frequent collaborations among a subset of authors. The network topology showed that some authors (e.g., Harris John E) served as central connectors, suggesting a leading role in thematic research development. However, the overall structure remains fragmented, with minimal inter-cluster connections, reflecting limited collaboration across different author communities. As author affiliations were not annotated in the visualization, no inference was made regarding institutional or national origin.

Country-clustering analysis revealed regional research partnerships and collaboration patterns (**Figure 5B**). The analysis revealed a strong Russia/Turkey/Spain collaboration in targeted therapy-induced vitiligo and USA/Philippines/Slovakia research networks emphasizing emerging therapies.

The country collaboration network revealed how nations worked together in vitiligo research (**Figure 5C**). Several countries stand out as major players. The USA is located at the center of many collaborations that connect researchers across continents. China has built strong partnerships with multiple countries, which reflects its growing research presence. European nations, such as Italy and Germany, have acted as regional bridges, linking European researchers while maintaining ties with American and Asian colleagues. India has carved out its own collaborative niche, particularly for Asian research. Each country brought its own strengths to these partnerships: some focused on building regional networks, whereas others cast a wider net globally.

The institutional collaboration map reveals distinct collaboration patterns of how research centers are connected globally (**Figure 5D**). France sat at the heart of the network, flanked by American and Egyptian institutions. The University of Massachusetts has built a particularly strong bridge with French researchers, as shown by the blue collaboration zone. However, the Chinese and Iranian research clusters stood somewhat apart from this central hub, suggesting that they focused more on building domestic networks. This pattern revealed that both the success of trans-Atlantic partnerships and untapped opportunities, bringing these isolated research communities into closer collaboration, could unlock new possibilities for advancing worldwide vitiligo treatment.

### 3.6 Conceptual structure and citation networks

**Figure 6:**
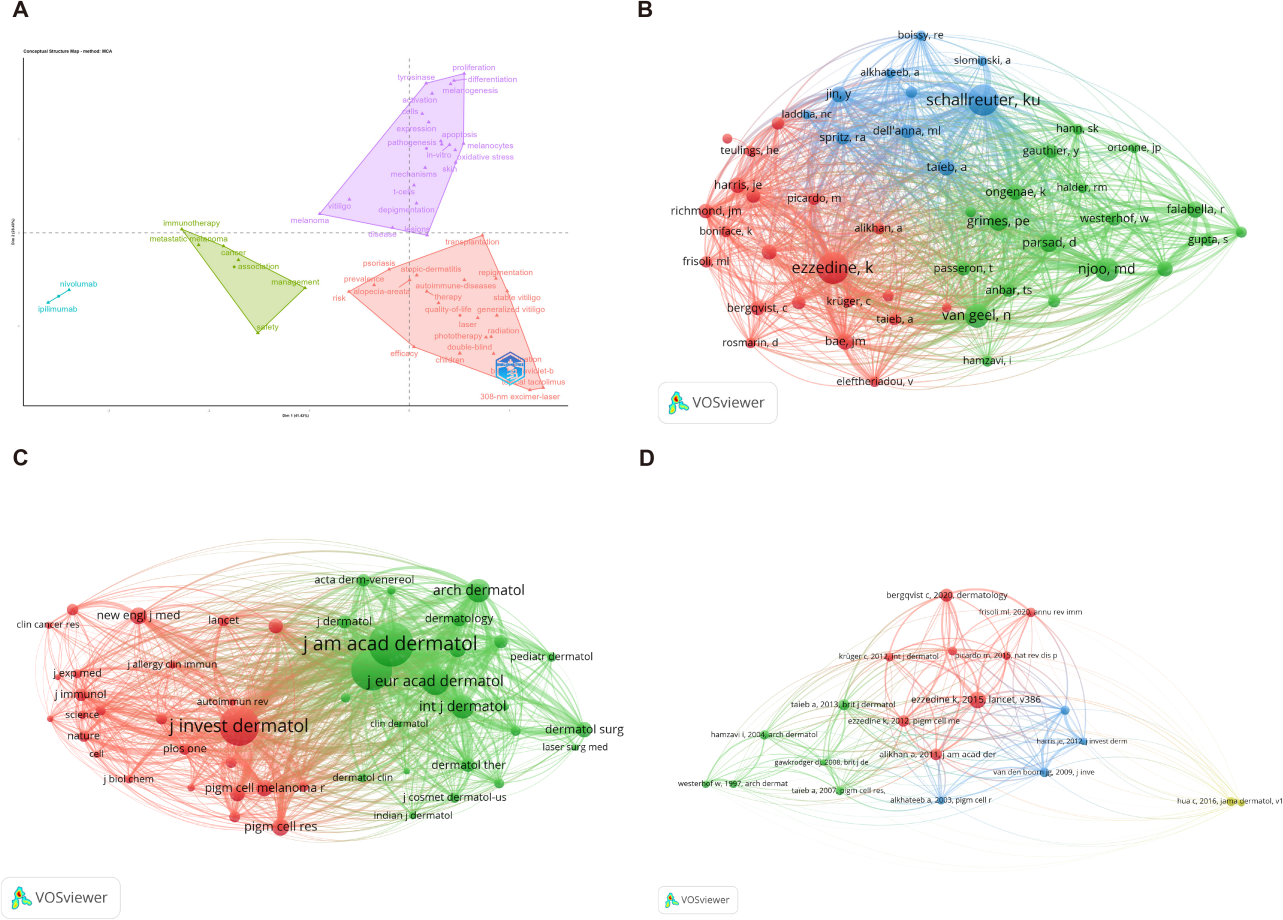
Multiple correspondence and co-citation analysis. **(A)** Multiple correspondence analysis (MCA) showing the relationships between the keyword items. **(B)** Author co-citation network identifying influential researchers and their research communities, with node size representing citation frequency. **(C)** Journal co-citation network showing the most frequently co-cited journals in vitiligo treatment research, with dermatology journals *J am acad dermatol* and *J invest dermatol* forming central clusters. **(D)** Reference co-citation network identifying landmark publications and their relationships, with node size representing citation frequency and edge thickness representing co-citation frequency.

Multiple correspondence analysis (MCA) identified three distinct research dimensions (**Figure 6A**). The purple triangular region in the upper portion contains the pathogenesis-related terms. The green triangular area on the left includes the “immunotherapy” and “metabolic” terms. The red triangular region on the right encompasses the clinical treatment approaches.

The author’s co-citation network revealed four major research hubs (**Figure 6B**). “Ezzedine, K” appeared as the largest central node, indicating a high co-citation frequency. “Schallreuter, Ku” emerged as another prominent node in the upper right area. Two additional major nodes were visible in the network, representing other influential researchers in the field. The network showed multiple colored clusters with dense interconnections between the leading authors.

The journal co-citation network demonstrated the interdisciplinary nature of vitiligo research (**Figure 6C**). The density map featured “*Brit J Dermatol*” and “*J Invest Dermatol*” as central nodes. Other important dermatology journals, including “*J Am Acad Dermatol*”, “*Int J Dermatol*”, “*J Eur Acad Dermatol & Venereol*”, and “*Arch Dermatol*” were also visible in the network, indicating their significant contributions to the field’s knowledge base.

The document co-citation network mapped intellectual structure through key publications (**Figure 6D**). Several landmark papers were clearly identifiable, including “Ezzedine K, 2015”, “Harris JE, 2012” and other influential works with their publication years. The network shows how these seminal papers connect to form knowledge clusters, with connection lines indicating co-citation relationships that reveal the field’s intellectual evolution and foundational literature.

### 3.7 Thematic evolution and research trends

**Figure 7:**
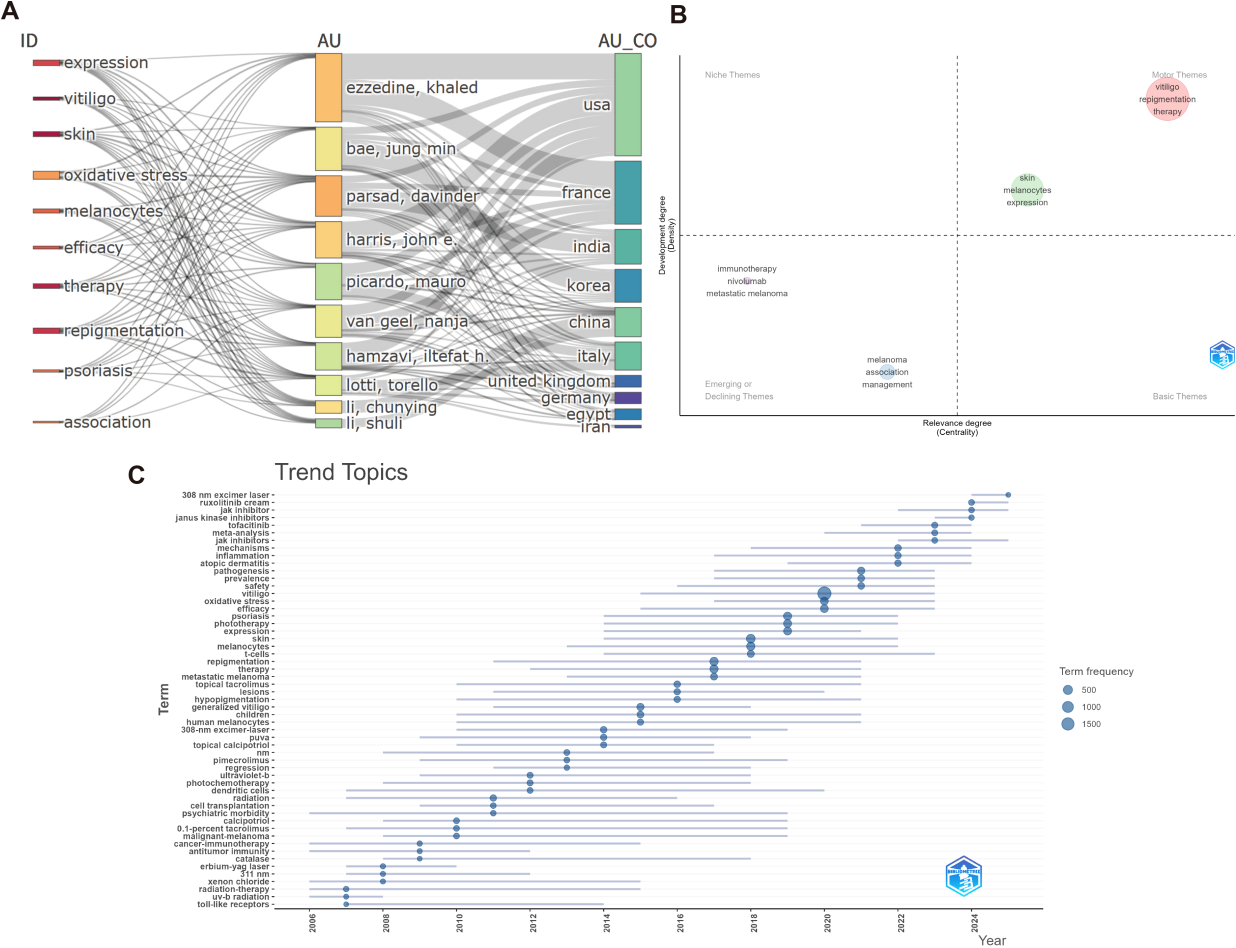
Thematic evolution and strategic positioning. **(A)** Three-field plot diagram: Integrating Keyword Plus (ID), Author (AU), and Country (CO) fields, illustrating the shifts, mergers, and associations of core research themes with key contributors and their affiliated countries. **(B)** Theme centrality map positioning research themes based on their centrality (relevance to the field) and density (internal development). Motor Themes are well-developed and central; Niche Themes are peripheral but highly developed; Basic Themes are central but underdeveloped; Emerging Themes are peripheral and underdeveloped. **(C)** Temporal trend map showing keyword evolution from 2005 to 2025. The timeline reveals a clear transition from empirical to mechanism-based treatment approaches.

The three-field plot diagram illustrates the relationships between keywords plus terms (ID), authors (AU), and their countries (AU_CO) (**Figure 7A**). Keywords Plus is a distinctive feature of the Web of Science database that automatically extracts keywords from the titles of cited references. These terms often reflect the core concepts and developmental trends of research (23). The visualization showed how specific Keyword Plus terms (extracted from reference titles) connected to particular researchers and their geographic locations. The flow patterns revealed how research themes, as captured by Keyword Plus analysis, were distributed across different authors and countries, demonstrating the global landscape of vitiligo treatment research expertise and thematic focus areas.

Thematic centrality analysis categorizes research themes into four quadrants on the basis of their centrality (interconnection with other themes) and density (internal coherence) (24, 25) (**Figure 7B**). Three core themes—vitiligo, repigmentation, and therapy—occupied the motor theme quadrant (upper right), demonstrating their role as primary drivers of field development, with strong interconnections and internal coherence. The Emerging or Declining Themes quadrant (lower left) contains immunotherapy and metastatic melanoma-related research, which are closely interconnected, as melanoma immunotherapy (particularly immune checkpoint inhibitors) can induce vitiligo-like depigmentation as an immune-related adverse event (26, 27). This positioning reflects the emerging recognition of treatment-induced vitiligo in the context of cancer immunotherapy and represents a growing research area that bridges oncology and dermatology. Notably, both Niche Themes and Basic Themes remained empty, indicating a polarized research landscape in which the field focused on well-developed core therapeutic concepts while actively exploring emerging cross-disciplinary approaches, reflecting a mature domain with clear priorities and strategic innovation directions.

The temporal trend map of keywords (**Figure 7C**) provides a nuanced view of the research trajectory of vitiligo treatment over the past two decades. Early in this period, interest focused on classical interventions such as *UV-B radiation*, *tacrolimus*, and *calcipotriol*. These terms showed prolonged activity for several years, suggesting their foundational role in the initial development of treatment protocols. As the timeline advances, there has been a shift toward immunologically driven mechanisms, including *oxidative stress*, *T cells*, and *inflammation, has occurred*. Notably, *cell transplantation*, a key therapeutic strategy for stable vitiligo, especially in refractory individuals, has attracted increasing attention over an extended period. In recent years, highly specific molecular targets such as *JAK inhibitors*, *tofacitinib*, and *ruxolitinib cream* have emerged as high-frequency options, reflecting the growing dominance of targeted immunotherapy in clinical research. This thematic evolution highlights the field’s transition from broadly immunosuppressive treatments to precision-driven approaches, grounded in molecular immunology. The increasing prominence of terms associated with immune modulation signaled a progressive refinement in the understanding of vitiligo pathogenesis and narrowed the focus on personalized therapeutic strategies.

### 3.8 Citation impact and influential publications

**Figure 8:**
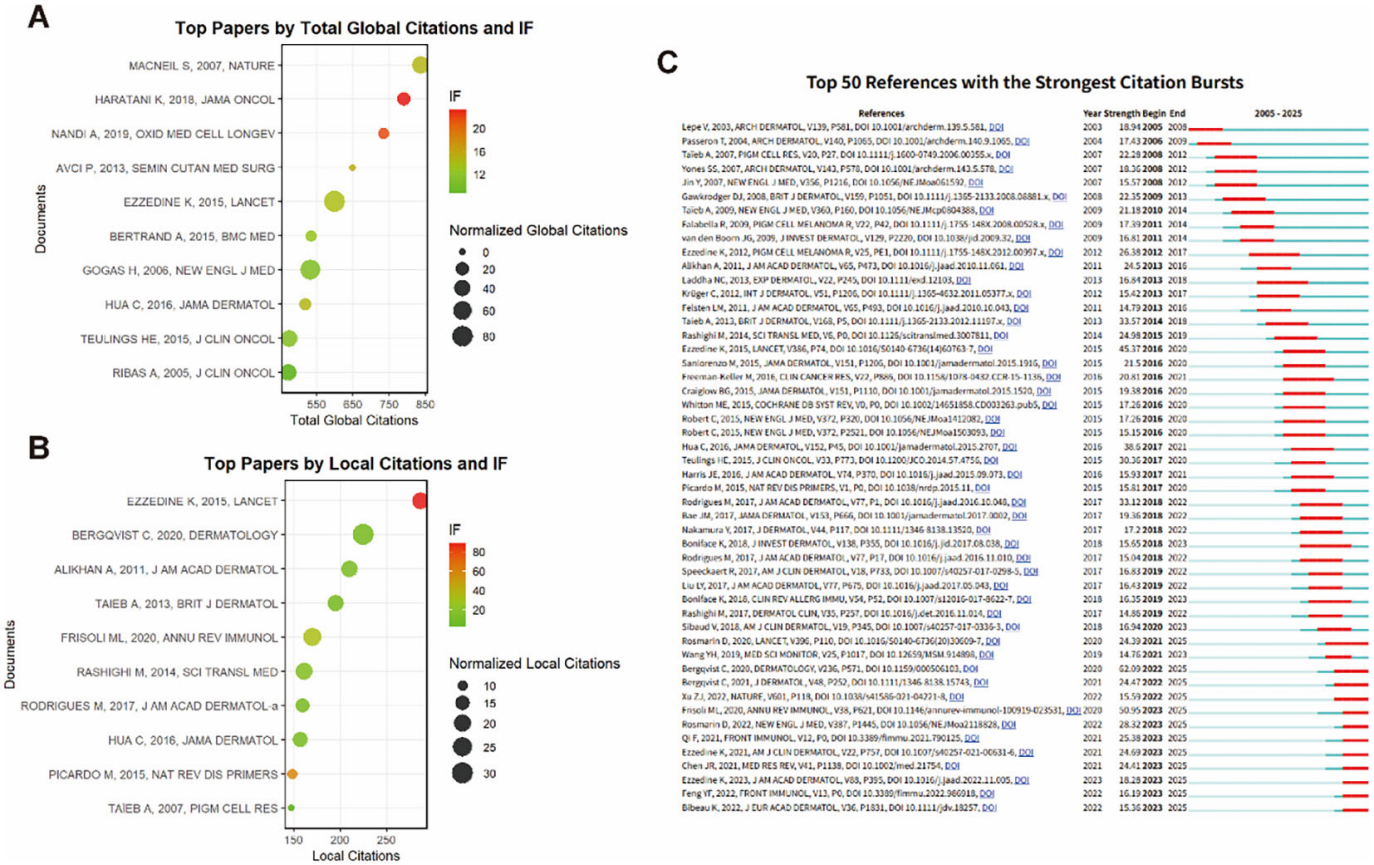
Citation analysis. **(A)** Top 10 globally cited articles ranked by total citations (bubble size) and journal impact factor **(IF)** (color gradient), showing landmark publications that have shaped the field. High-impact journals (Nature, Lancet, New Engl J Med) contain highly cited papers, indicating that vitiligo research has achieved visibility in top-tier journals. **(B)** Top 10 locally cited articles within the vitiligo research community, showing publications with high impact within the specialized field but potentially lower global citations. **(C)** Top 50 references with the strongest citation bursts, indicating publications that significantly influenced research direction during specific time periods. Red bars indicate burst periods.

The bubble chart of the top 10 globally cited papers shows the relationship between citation count and the journal impact factor (**Figure 8A**). High-impact publications have demonstrated sustained influence in the field, with several landmark studies maintaining strong citation patterns over multiple years. Local citation analysis revealed that publications had a significant impact on the vitiligo research community (**Figure 8B**). The bubble chart displays locally influential papers that have shaped research directions within a specialized field.

Citation burst analysis identified the top 50 references with the strongest citation bursts, indicating publications that significantly shaped research direction (**Figure 8C**). The data identified landmark articles with significant citation bursts, including Harris (2016), Picardo (2015, 2018) and Ezzedine (2015, 2023). These studies are instrumental in reshaping vitiligo treatment pathways by providing mechanistic insights and validating the immune targets.

Author productivity and impact analyses (**Table 4**) revealed that Harris John E from the University of Massachusetts had the highest h-index, followed by other prominent researchers in the field. The rankings reflect both the publication volume and citation impact. The results revealed a marked discrepancy between publication volume and impact metrics. Although some authors exhibited a high output, their citation metrics were relatively low, indicating varying degrees of influence. Conversely, several authors with fewer publications achieved high h- and g-indices, suggesting a sustained scholarly impact and recognition. This highlights the importance of evaluating both quantity and quality of academic productivity.

**Table 4:** Ranking of academic quantitative indicators of the top 10 authors.

Author	h-index	g-index	m-index	TC	NP	PY_start
HARRIS JOHN E.	26	37	1.857	3586	37	2012
EZZEDINE KHALED	23	46	1.643	3284	46	2012
TAIEB ALAIN	19	26	1	2149	26	2007
LI CHUNYING	18	36	1.2	1609	36	2011
PARSAD DAVINDER	18	34	0.9	1226	51	2006
PICARDO MAURO	18	28	0.947	2108	28	2007
BAE JUNG MIN	17	30	1.545	935	32	2015
HAMZAVI ILTEFAT	16	26	0.842	1724	26	2007
LI SHULI	16	27	1.231	1088	27	2013
VAN GEEL NANJA	16	32	1.067	1512	32	2011

TC, Total Citations; NP, Number of Papers; PY, Publication Year.

An analysis of the top 20 most-cited publications revealed milestone contributions that spanned the entire research period (**Table 5**). These papers, although not always the most cited globally, played a key role in shaping the discourse within this specific field. Many of these highly cited studies were related to treatment guidelines, immune mechanisms, and targeted therapy trials, particularly those discussing JAK inhibitors and phototherapy. The high citation frequency of these studies underscores their foundational relevance in subsequent publications in this domain.

**Table 5:** Ranking of the top 20 cited papers and detailed information.

Paper	DOI	Total citations	TC per year	Normalized TC
MACNEIL S, 2007, NATURE	10.1038/nature05664	837	44.05	14.62
HARATANI K, 2018, JAMA ONCOL	10.1001/jamaoncol.2017.2925	790	98.75	23.18
NANDI A, 2019, OXID MED CELL LONGEV	10.1155/2019/9613090	734	104.86	21.95
AVCI P, 2013, SEMIN CUTAN MED SURG	NA	648	49.85	15.50
EZZEDINE K, 2015, LANCET	10.1016/S0140-6736(14)60763-7	599	54.45	13.84
BERTRAND A, 2015, BMC MED	10.1186/s12916-015-0455-8	534	48.55	12.34
GOGAS H, 2006, NEW ENGL J MED	10.1056/NEJMoa053007	532	26.60	10.72
HUA C, 2016, JAMA DERMATOL	10.1001/jamadermatol.2015.2707	517	51.70	14.49
TEULINGS HE, 2015, J CLIN ONCOL	10.1200/JCO.2014.57.4756	474	43.09	10.96
RIBAS A, 2005, J CLIN ONCOL	10.1200/JCO.2005.01.109	471	22.43	8.68
ALIKHAN A, 2011, J AM ACAD DERMATOL	10.1016/j.jaad.2010.11.061	467	31.13	12.62
SIBAUD V, 2018, AM J CLIN DERMATOL	10.1007/s40257-017-0336-3	453	56.63	13.29
BERGQVIST C, 2020, DERMATOLOGY	10.1159/000506103	453	75.50	17.69
PAULOS CM, 2007, J CLIN INVEST	10.1172/JCI32205	451	23.74	7.88
TAÏEB A, 2007, PIGM CELL RES	10.1111/j.1600-0749.2006.00355.x	363	19.11	6.34
DAMSKY W, 2017, J AM ACAD DERMATOL	10.1016/j.jaad.2016.12.005	356	39.56	9.07
SANLORENZO M, 2015, JAMA DERMATOL	10.1001/jamadermatol.2015.1916	354	32.18	8.18
WANG PF, 2017, FRONT PHARMACOL	10.3389/fphar.2017.00730	344	38.22	8.76
RASHIGHI M, 2014, SCI TRANSL MED	10.1126/scitranslmed.3007811	341	28.42	13.92
FRISOLI ML, 2020, ANNU REV IMMUNOL	10.1146/annurev-immunol-100919-023531	326	54.33	12.73

TC, Total Citations.

Although some highly cited articles referred to vitiligo as an immune-mediated reaction in unrelated conditions (highlighted in **Supplementary Table S2**), their inclusion underlined the shared molecular mechanisms across immune disorders. Such cross-pollination of findings might inspire novel therapies through drug repurposing and systems-level modeling. These citation bursts also indicate where paradigm shifts had occurred and where the clinical focus might intensify in future trials.

### 3.9 Analysis of randomized controlled trials

After screening, 249 randomized controlled trials (RCTs) were identified from the PubMed database, showing an annual growth rate of 6.83% (**Supplementary Figure S3A**). An analysis of the corresponding authors’ countries revealed that Egypt, India, China, Iran, and France were the top five contributors (**Supplementary Figure S3B**). Among all the authors, Parsad D participated in the largest number of clinical trials (**Supplementary Figure S3C**) and was also the most prolific author in the Scopus dataset (**Supplementary Figure S4**). Regarding publication sources, dermatology-focused journals such as *Dermatologic Therapy*, *Journal of Cosmetic Dermatology*, and *Journal of the American Academy of Dermatology* were the most frequently selected outlets (**Supplementary Figure S3D**).

**Figure 9:**
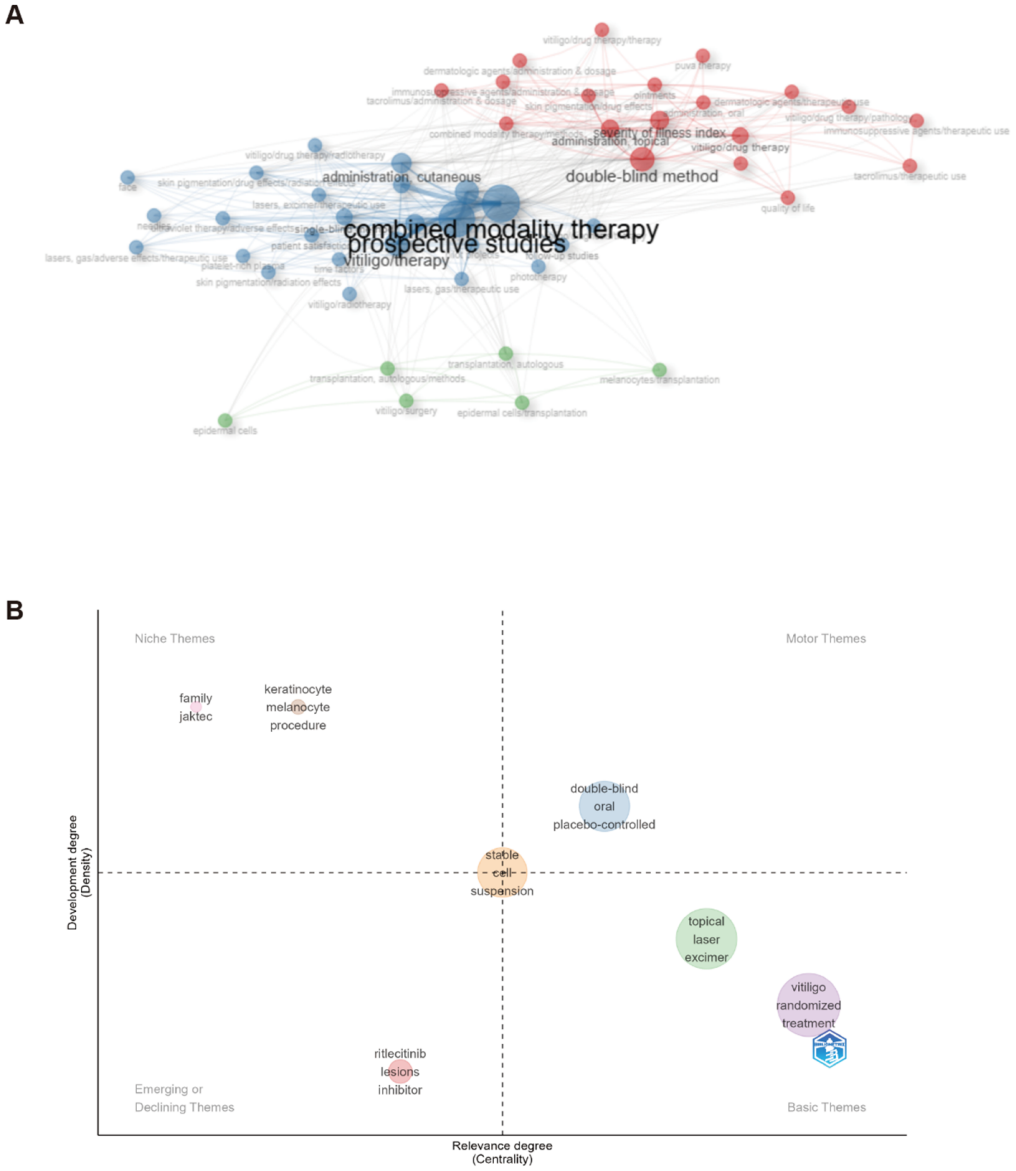
Randomized controlled trials analysis. **(A)** Co-occurrence network of terms from 249 Randomized Controlled Trials (RCTs) identified in PubMed, showing three major research themes. Node size represents term frequency. **(B)** Thematic map positioning RCT research themes based on centrality and density.

The Bibliometrix analysis further identified three major research themes: (1) pharmacological therapies (red cluster), (2) surgical interventions (green cluster), and (3) phototherapy-based physical treatments (blue cluster) (**Figure 9A**). Thematic map analysis also revealed that JAK family inhibitors, particularly the JAK3/TEC inhibitor ritlecitinib, are emerging as a key focus in ongoing clinical trials (**Figure 9B**).

## 4 Discussion

### 4.1 Increasing research attention reflects clinical unmet needs in autoimmune dermatology

Vitiligo affects 0.5–2% of the global population and often begins before the age of 30. This causes visible progressive depigmentation, which affects self-esteem and social functioning (28). Its unpredictable course, high recurrence rate, and limited curative therapies make it a challenging autoimmune disorder to manage. For many years, treatment options have been limited to corticosteroids and phototherapy, with variable results and frequent relapse.

Over the past two decades, vitiligo research has experienced steady expansion, with a compound annual growth rate of 7.27%. Based on observed publication trends, research activity can be broadly described as evolving from an initial period of gradual expansion toward a phase of more accelerated output in recent years. These temporal patterns appear to coincide with major advances in the understanding of vitiligo immunopathogenesis and the emergence of immune-targeted therapeutic approaches (5). The steady growth phase (2005–2010) laid foundational groundwork: topical calcineurin inhibitors, notably tacrolimus and pimecrolimus, became established as standard treatments, while narrowband UV-B phototherapy protocols were refined and standardized (29). This was followed by an accelerated growth period (2011–2018) defined by transformative mechanistic insights: researchers identified the JAK-STAT pathway as central to vitiligo pathogenesis and characterized key inflammatory mediators such as IFN-γ and CXCL10 (30), complemented by critical immunological discoveries including IFN-γ-driven disease progression and the role of CD8+ resident memory T cells in sustaining melanocyte destruction (31). Most recently, the rapid expansion phase (2019–2025) has seen the clinical translation of these foundational findings: JAK inhibitors have emerged as viable therapeutics, with the FDA approval of topical ruxolitinib in 2022 marking a watershed moment (6, 22). This approval not only validated the immune-targeted therapeutic paradigm but also spurred renewed interest in JAK-STAT pathway inhibition, reflecting a broader paradigm shift—reclassifying vitiligo as an immune-mediated disorder rather than a purely cosmetic concern. Collectively, this shift has significantly expanded the research landscape and therapeutic arsenal, with concurrent advancements in precision medicine and novel biologics further enriching the field (32).

### 4.2 The need for improved international collaboration in translational immunology

Our bibliometric findings reveal that, while China, the United States, and India lead in terms of publication volume, international collaboration remains limited. China had the highest number of articles, but a low MCP ratio (7.3%), indicating that most studies were conducted domestically. In contrast, European countries such as France and the UK presented higher MCP ratios, suggesting stronger international networks (33).

Interestingly, although the Egyptian Knowledge Bank (EKB) has the highest institutional output, it acts mainly as a national publication platform, and its research impact requires further evaluation. Traditional academic institutions such as Harvard University and INSERM (France) have shown sustained output and collaboration. Notably, the University of Massachusetts did not appear in the top 10 (top 30; see **supplementary Table S3**); however, Harris John E was identified as the most influential author by the h-index and citation metrics. This finding illustrates that institutional impact cannot be inferred solely from publication counts; individual leadership plays a key role in shaping the field.

### 4.3 From empirical regimens to immune-targeted precision therapies

Our keyword co-occurrence and thematic evolution analyses reflected a clear shift in research focus from conventional modalities, such as phototherapy and topical agents, toward molecular-targeted therapies. JAK inhibitors, particularly tofacitinib and ruxolitinib, were among the most recently cited keywords. This shift mirrors a broader trend toward immune modulation and precision therapy in autoimmune dermatology (34).

Earlier treatment approaches often targeted inflammation in a nonspecific manner. In contrast, accumulating mechanistic studies have clarified the central involvement of interferon-γ (IFN-γ)–driven signaling, chemokine (C-X-C motif) ligand 10 (CXCL10), and tissue-resident memory T (Trm) cells in sustaining melanocyte loss and disease persistence (35). These findings provide a coherent biological framework linking chronic immune activation to both lesion maintenance and relapse.

Within this mechanistic context, inhibition of the JAK–STAT pathway represents a rational strategy for disrupting pathogenic cytokine signaling rather than indiscriminately suppressing immune function. Importantly, this paradigm shift reflects advances in understanding disease pathophysiology and immune regulation, rather than definitive conclusions regarding clinical superiority. As such, immune-targeted precision therapies should be viewed as the logical extension of mechanistic insight, setting the stage for their integration into broader clinical treatment strategies discussed below.

### 4.4 Cross-disciplinary integration: linking autoimmunity, oncology, and immunotherapy

The dual-map overlay and multiple correspondence analysis (MCA) results suggest that vitiligo research is becoming increasingly interdisciplinary, with dermatology intersecting with immunology, oncology, and molecular biology. This disciplinary convergence reflects a fundamental shift in how vitiligo is conceptualized and studied. Historically, vitiligo was primarily viewed as a dermatological condition; however, contemporary research increasingly recognizes its immunological and oncological dimensions.

The connection between vitiligo and oncology is particularly illuminating. Research on immune checkpoint inhibitors (ICIs) in melanoma patients has revealed important mechanistic insights into vitiligo pathogenesis. ICIs, which are designed to enhance anti-tumor immunity by blocking inhibitory pathways, frequently induce vitiligo-like depigmentation as an adverse effect. This paradoxical phenomenon—where cancer immunotherapy triggers melanocyte destruction—reinforces the shared autoimmune underpinnings between cancer therapy and pigment loss, and has become a critical area of investigation (26). The observation that ICIs can trigger vitiligo-like reactions has profound implications for understanding the disease mechanism and has catalyzed a productive cross-pollination of knowledge between dermatological and oncological research communities.

Citation burst analysis further highlights the foundational studies that have shaped the field’s intellectual landscape. Many high-impact papers have focused on the mechanisms of depigmentation, the psychosocial burden of disease, and emerging therapeutic approaches. These seminal studies remain central references for both clinical trials and basic research, indicating their enduring influence on the field’s trajectory. The persistence of citations to these foundational works demonstrates their continued relevance despite the rapid evolution of therapeutic options.

The analysis of citation pathways reveals distinct patterns of knowledge flow within and across disciplines. Several major citation pathways emerged from this analysis, with dermatology specialty journals (e.g., *J Eur Acad Dermatol & Venereol*) frequently citing high-impact general medical journals (e.g., *New Engl J Med*). These findings not only indicate the clinical relevance of vitiligo research but also reinforce the interdisciplinary nature of modern vitiligo scholarship. This pattern mirrors similar transitions observed in the study of other inflammatory skin diseases such as psoriasis and atopic dermatitis, where dermatological research has increasingly engaged with immunological and systemic perspectives (36, 37).

Notably, oncology journals (e.g., *Pigm Cell Melanoma R*) and immunotherapy literature have also emerged as significant contributors to the vitiligo research discourse. This cross-disciplinary citation pattern has strengthened considerably with the increased recognition of vitiligo-like reactions in patients with cancer receiving immunotherapy. The growing volume of citations linking vitiligo research to oncology and immunotherapy literature suggests that the field has recognized the value of integrating insights from cancer biology and immunotherapy development. This interdisciplinary engagement has created new opportunities for understanding vitiligo’s pathogenesis and identifying novel therapeutic targets.

### 4.5 From publication trends to clinical evidence: assessing translational immunology pipeline

Rather than establishing causality, the integration of RCT data provides clinical context for interpreting publication trends observed in the broader literature. However, limitations in the current clinical evidence base remain evident. While JAK inhibitors have rapidly translated into clinical practice following the FDA approval of ruxolitinib cream in 2022, long-term safety data beyond 12 months remain limited in RCT literature. Similarly, pediatric populations appear underrepresented in clinical trials, despite vitiligo often beginning before the age of 30 (38). The predominance of dermatology-focused journals (*Dermatologic Therapy, Journal of Cosmetic Dermatology, Journal of the American Academy of Dermatology*) as publication outlets for RCTs suggests that vitiligo clinical research remains primarily within the dermatology community, with limited cross-disciplinary engagement in high-impact general medical journals. These gaps should inform the priorities and publication strategies of future clinical trials.

The geographical distribution of RCTs provided interesting insights. Egypt, India, China, Iran, and France emerged as the top five contributors to the RCT output. This pattern differs somewhat from the overall publication rankings, which are dominated by China and the USA. The prominence of Egypt and India in RCT contributions, despite their lower positions in total publication volume, suggests active clinical trial participation and patient recruitment capacity in these regions with a high disease burden (38–40). Among the individual researchers, Parsad D from India participated in the largest number of clinical trials and was also identified as the most prolific author in the Scopus dataset, exemplifying how individual leadership can drive both research output and clinical translation.

Our RCT analysis revealed three major research themes that aligned well with the keyword co-occurrence patterns identified in the broader literature: pharmacological therapies, surgical interventions, and phototherapy-based physical treatments. Notably, JAK inhibitors have emerged as a dominant focus in recent RCTs, with ritlecitinib (a JAK3/TEC inhibitor) identified as an emerging key focus in ongoing clinical trials (41). This temporal concordance between citation bursts for JAK inhibitors (2019–2022 in our keyword analysis) and RCT activity suggests that bibliometric indicators may reflect trends associated with emerging therapeutic directions (42), and is consistent with the hypothesis that vitiligo research is experiencing active translational development in which mechanistic discoveries are efficiently channeled into clinical testing.

The proportion of RCTs (7.1%) in vitiligo treatment research aligns with patterns observed in other chronic dermatological conditions, suggesting that this reflects the typical balance between mechanistic research and clinical evidence generation in dermatology, rather than representing a unique evidence gap. However, the underrepresentation of long-term safety data (>12 months) and pediatric populations within these RCTs remains a concern specific to vitiligo, given its early onset and chronic nature.

The analysis of 249 randomized controlled trials provided a crucial validation of the thematic shifts identified through bibliometric analysis. RCTs represented 7.1% of the total publications (249 of 3, 483), a proportion consistent with other dermatological conditions (43) but highlighted the predominance of observational studies and mechanistic research in this field. The annual growth rate of RCTs (6.83%) slightly lagged behind the overall publication growth (7.27%), suggesting that while research interest is expanding rapidly, the generation of high-quality clinical evidence requires more time and resources.

### 4.6 Therapeutic landscape and evolving treatment strategies for vitiligo

Current treatments for vitiligo are mainly directed at suppressing autoimmune-mediated melanocyte damage, stabilizing disease activity, and inducing repigmentation (44)(**Table 6**). For localized non-segmental vitiligo, topical corticosteroids and topical calcineurin inhibitors remain first-line options, particularly for the face and intertriginous areas (45), where efficacy is generally favorable and tolerability is acceptable.

**Table 6:** Current and emerging therapeutic strategies for vitiligo.

Category	Therapy type	Representative examples	Main clinical role	Key mechanism	Evidence stage
Current therapies	Topical immunomodulators	Corticosteroids; calcineurin inhibitors	Localized non-segmental vitiligo	Suppression of cutaneous T-cell–mediated inflammation	Guidelines
	Topical targeted therapy	Ruxolitinib cream	Facial and limited BSA vitiligo	JAK1/2 inhibition of IFN-γ signaling	FDA approved
	Phototherapy	NB-UVB; excimer	Generalized vitiligo; repigmentation	Immunomodulation; activation of follicular melanocytes	High-level evidence
	Systemic immunosuppression	Corticosteroid minipulse; methotrexate	Active or progressive disease	Broad immune suppression	Consensus/limited
	Surgical/depigmentation approaches	MKTP; tissue grafts; monobenzone	Stable or extensive refractory disease	Melanocyte replacement or elimination	Selected clinical use
Emerging therapies	Systemic JAK inhibition	Ritlecitinib; upadacitinib	Active or extensive vitiligo	IFN–JAK–STAT pathway blockade	Phase II trials
	TRM-targeted immunotherapy	Anti-IL-15; anti-CD122	Relapse prevention	Disruption of tissue-resident memory T cells	Early clinical
	Targeted IFN-γ modulation	Skin-restricted IFN-γ antibodies	Precision cytokine inhibition	Local IFN-γ blockade	Preclinical
	Oxidative stress/senescence targeting	Nrf2 activators; senolytics	Microenvironment restoration	Suppression of oxidative stress–induced senescence	Preclinical
	Regenerative and delivery strategies	MSCs; PRP; exosomes; nanocarriers	Tissue repair and enhanced drug delivery	Microenvironment remodeling; improved penetration	Early translational
	RNA interference–based therapy	siRNA approaches	Pathway-specific gene silencing	Post-transcriptional inhibition	Preclinical

BSA, body surface area; IFN, interferon; JAK, Janus kinase; MKTP, melanocyte–keratinocyte transplantation procedure; NB-UVB, narrowband ultraviolet B; PRP, platelet-rich plasma; RNAi, RNA interference; TRM, tissue-resident memory T cells.

Phototherapy remains the cornerstone treatment for patients with more extensive disease. Narrowband ultraviolet B (NB-UVB) is the most widely used modality and promotes repigmentation through both immune modulation and activation of follicular melanocyte reservoirs (29). Targeted ultraviolet B phototherapy, including 308 nm excimer laser or lamp, is commonly used for localized or treatment-resistant lesions. By selectively delivering high-dose UVB to depigmented skin while sparing surrounding normal tissue, excimer therapy improves precision and reduces cumulative UV exposure (46). Nevertheless, responses remain highly site dependent, with acral and mucosal areas responding poorly.

For patients with rapidly progressive or highly active vitiligo, systemic immunosuppressive strategies are often required to achieve disease stabilization (47). Intermittent systemic corticosteroid minipulse regimens are widely applied in clinical practice, frequently in combination with phototherapy (48). Conventional systemic immunosuppressants, such as methotrexate (49) or cyclosporine (50), may be considered in selected cases, particularly when corticosteroids are contraindicated, although supporting evidence remains limited. In stable vitiligo refractory to medical therapy, surgical approaches such as melanocyte–keratinocyte transplantation can induce repigmentation (51), whereas depigmentation therapy with monobenzone is reserved for extensive disease with minimal residual normal pigmentation (52).

Despite their clinical utility, most conventional therapies are non-specific and primarily target inflammation rather than the mechanisms underlying disease persistence and relapse. The approval of topical ruxolitinib provides direct clinical evidence that inhibition of the interferon-γ–JAK–STAT pathway can restore pigmentation in non-segmental vitiligo (53). Oral JAK inhibitors are now under investigation for patients with active or extensive disease (54), underscoring their translational relevance while also raising important questions regarding long-term safety, durability of response, and optimal patient selection.

IFN-γ plays a central role in vitiligo pathogenesis; however, systemic neutralization is clinically impractical due to the risk of broad immunosuppression (55). Recent studies have shown that skin-restricted approaches, such as a keratinocyte-tethered IFN-γ antibody, can effectively ameliorate vitiligo in mouse models while minimizing off-target immune effects, supporting the feasibility of tissue-targeted cytokine blockade (56).

Beyond direct immune suppression, increasing attention has been directed toward factors that contribute to disease chronicity and relapse. Tissue-resident memory T cells are now recognized as key drivers of relapse, leading to the exploration of IL-15–dependent survival pathways as therapeutic targets. At the same time, cellular senescence in keratinocytes (57), melanocytes (57), and dermal fibroblasts (58, 59) has emerged as a potential contributor to melanocyte dysfunction. Oxidative stress is a major trigger of senescence (60), suggesting that strategies aimed at reducing oxidative stress, activating antioxidant pathways such as Nrf2 (61), or selectively eliminating senescent cells may represent future treatment directions (62).

Additional emerging approaches focus on restoring the cutaneous microenvironment and improving therapeutic precision. Regenerative strategies, including mesenchymal stem cells, platelet-rich plasma, and stem cell–derived exosomes (63), aim to support melanocyte survival and tissue repair rather than simply suppress immune activity. Advances in local drug delivery, such as nanocarriers (64) and laser-assisted drug delivery (65), seek to enhance intradermal drug penetration while limiting systemic exposure. RNA interference–based therapies further expand this concept by enabling pathway-specific gene silencing (66, 67). Although these approaches are currently limited to preclinical or early translational studies, they highlight a gradual shift toward more precise and potentially disease-modifying treatment strategies for vitiligo.

### 4.7 Study limitations and future directions

This study had several limitations that should be acknowledged. First, despite incorporating multiple databases (WoSCC, Scopus, and PubMed), data harmonization presents challenges owing to differences in metadata formats, citation indexing, and author name disambiguation (68). Although we employed standardized tools (R bibliometrix) to minimize inconsistencies, some degree of variability was inevitable. Second, our analysis was restricted to English-language publications, which may have underrepresented research from non-English-speaking regions, particularly Latin America, Russia, and parts of Asia where vitiligo research is active. Future bibliometric studies could incorporate regional databases (e.g., CNKI for Chinese literature and SciELO for Latin American literature) to capture a more comprehensive global picture (69, 70).

Another limitation lies in the interpretation of institutional productivity. Aggregator platforms such as EKB were ranked highly owing to national funding policies, but their bibliometric weight may overrepresent collective rather than original academic output. Additionally, author influence and institutional output should be analyzed separately, as demonstrated by Harris.

Future research could apply machine learning to improve the cluster identification and trend forecasting. More refined bibliometric tools could also distinguish between therapeutic and mechanistic studies or separate adult and pediatric populations, which are currently indistinct in most studies.

## 5 Conclusions

This study offers a multi-database overview of research on vitiligo treatment, combining publication trends with available randomized controlled trial (RCT) evidence. Across databases, several consistent patterns emerge. Research output continues to grow steadily. China, the United States, and India remain the main contributors. At the same time, treatment-related studies show a gradual shift from conventional approaches toward immune-targeted therapies. The approval of topical ruxolitinib represents an important milestone in this context and coincides with increasing clinical trial activity focused on JAK inhibition. Nevertheless, the overall proportion of RCTs remains low, long-term safety data are still limited, and pediatric populations are underrepresented.

Beyond JAK inhibitors, recent studies have begun to explore additional therapeutic directions, including RNA interference–based strategies, skin-targeted IFN-γ modulation, advanced drug delivery systems, and regenerative cell-based approaches. Most of these strategies are still at an early stage, but they reflect ongoing efforts to improve treatment specificity and durability. Viewed together, these findings place current and emerging therapies within a broader research landscape and emphasize the continued need for well-designed clinical studies to address remaining gaps in vitiligo management.

## Data Availability

The original contributions presented in the study are included in the article/**supplementary material**. Further inquiries can be directed to the corresponding author.
