# Supplementary material for: From immunological mechanisms to targeted therapies: a bibliometric analysis of vitiligo treatment research (2005–2025)
Source: Front Immunol. 2026 Jan 14;16:1733857. doi: 10.3389/fimmu.2025.1733857 (PMC12847362; doi:10.3389/fimmu.2025.1733857)
Supplement: Supplementary file 1 [file DataSheet1.docx]

**
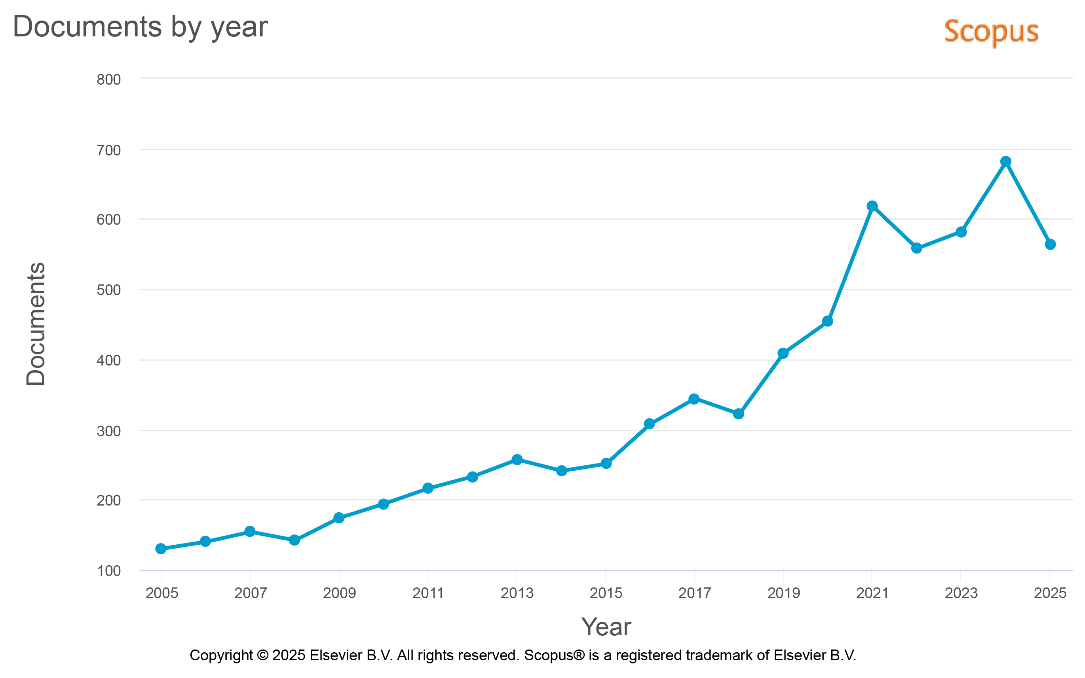
**

**Figure S1. Scopus validation of publication trends.** Annual vitiligo treatment publications from Scopus (2005–2025) showing sustained growth with three phases: steady (2005–2010), moderate (2011–2017), and rapid expansion (2018–2024, peak: 681 documents). Trend validates Web of Science findings, confirming robustness across databases.


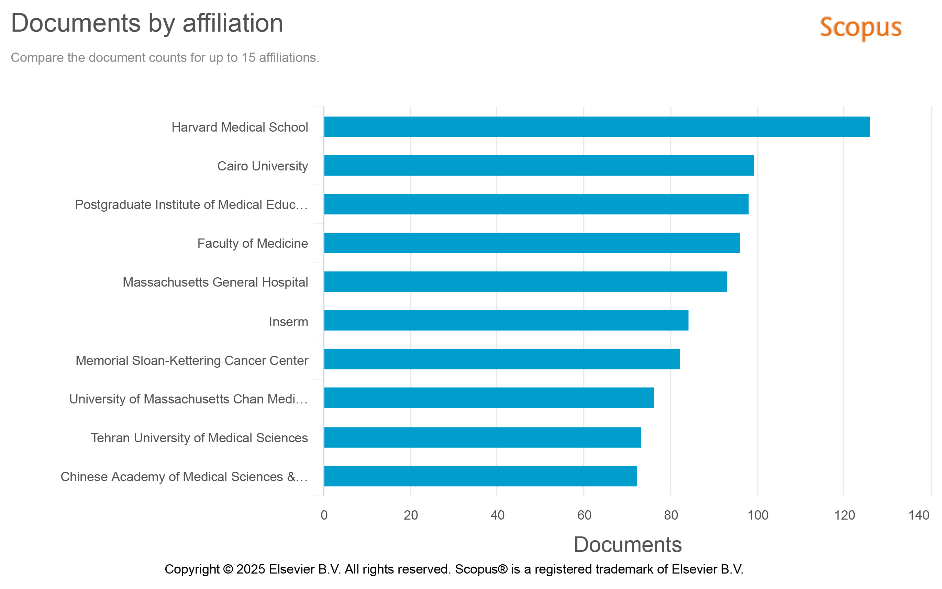


**Figure S2. Scopus validation of institutional contributions.** Top 10 institutions from Scopus led by Harvard Medical School (126 documents), Cairo University (99), and Postgraduate Institute of Medical Education (98). Rankings validate prominence of Harvard-affiliated institutions and contributions from Egypt, India, Iran, and China identified in Web of Science analysis.


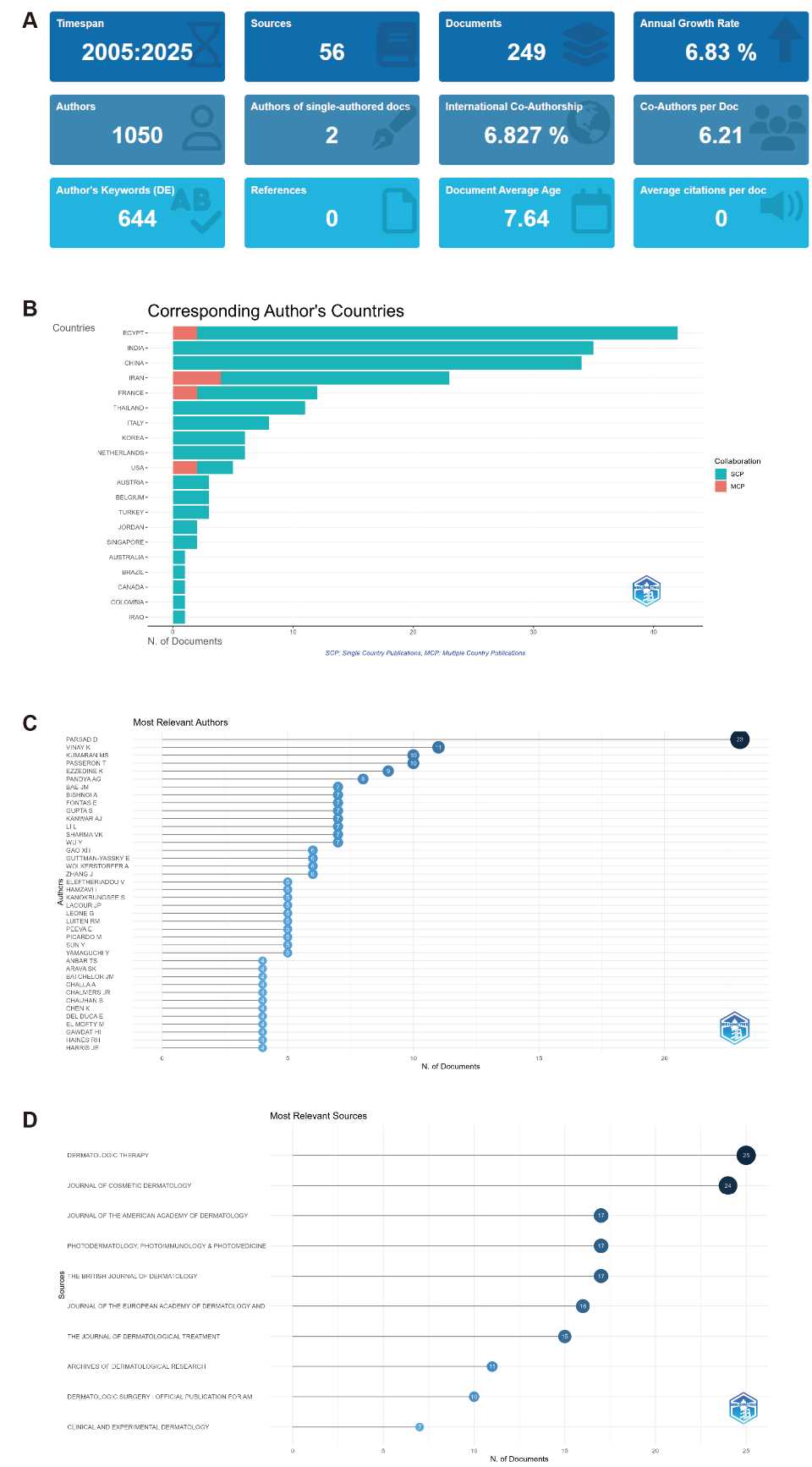


**Figure S3. PubMed RCT bibliometric overview using Bibliometrix (R package).** (A) Summary: 249 RCTs, 1,050 authors, 6.83% annual growth. (B) Geographic distribution: Egypt, India, China lead; limited international collaboration. (C) Top authors: Parsad D (23 publications), Vinay K (11), Kumaran MS (10). (D) Top journals: Dermatologic Therapy (25), Journal of Cosmetic Dermatology (24).

**
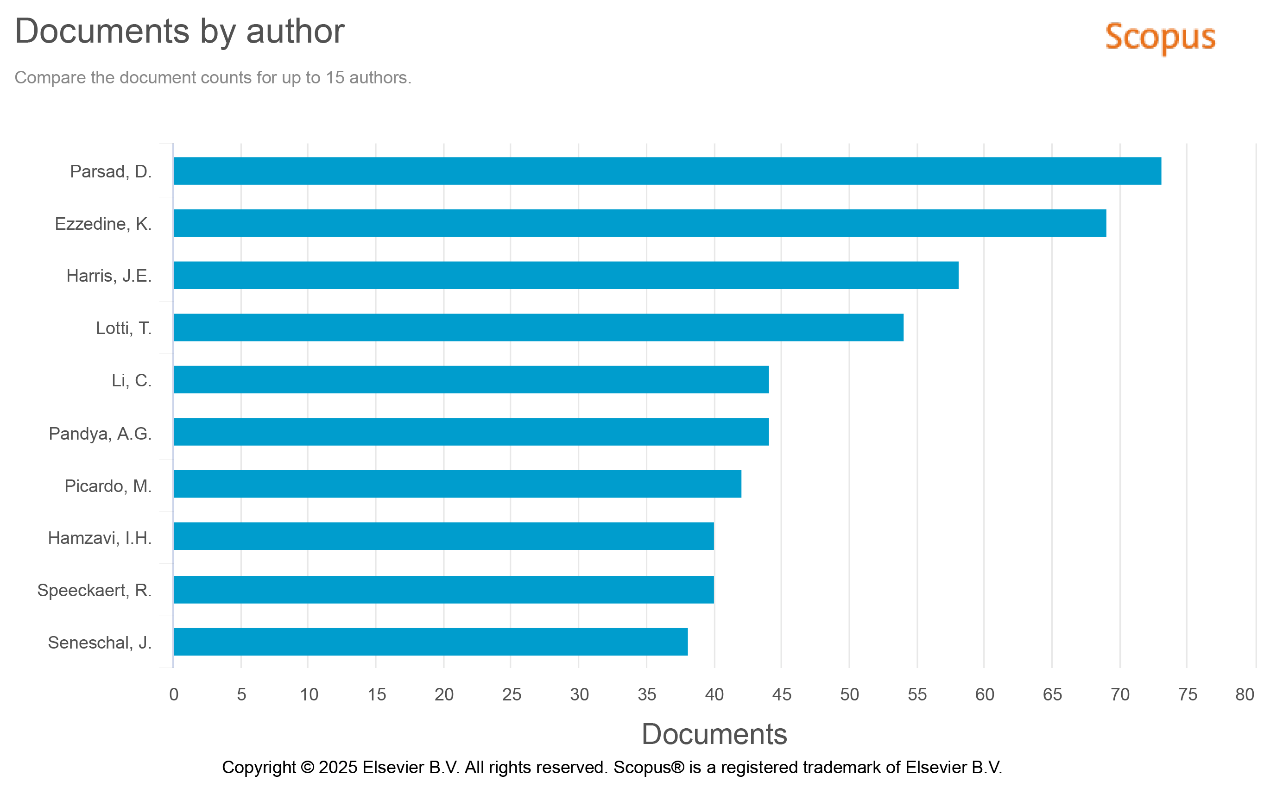
**

**Figure S4. Scopus validation of author contributions.** Top 10 authors from Scopus: Parsad D (~78 documents), Ezzedine K (~70), Harris JE (~60). Parsad D's leadership corroborates RCT analysis findings (28 RCTs, highest participation). Cross-database validation confirms reliability of author impact assessments.

**Table S1.** Inclusion and Exclusion Criteria for PubMed RCTs

| **Inclusion criteria** | **Exclusion criteria** |
| --- | --- |
| (1) randomized controlled trial design (parallel or split-body);  (2) patients diagnosed with vitiligo of any clinical subtype;  (3) evaluation of a therapeutic intervention aimed at disease stabilization or repigmentation, including topical, systemic, phototherapy-based, or surgical approaches;  (4) English-language, peer-reviewed publications published between January 1, 2005 and October 21, 2025; and  (5) availability of sufficient methodological and outcome information to permit data extraction. | (1) non-randomized or observational study designs;  (2) studies involving non-vitiligo populations or mixed populations without separate vitiligo data;  (3) interventions not intended for therapeutic management of vitiligo (e.g., purely mechanistic or diagnostic studies);  (4) absence of clinical efficacy or safety outcome reporting;  (5) non-English publications, conference abstracts, or protocol-only reports; and  (6) duplicate publications or interim analyses of the same trial. |

**Table S2.** The direct correlation to vitiligo treatment of Top 20 cited articles

| **Paper** | **Title** | **Relevant to vitiligo treatment (Y/N)** |
| --- | --- | --- |
| MACNEIL S, 2007, NATURE | Progress and opportunities for tissue-engineered skin | Y |
| HARATANI K, 2018, JAMA ONCOL | Association of Immune-Related Adverse Events With Nivolumab Efficacy in Non–Small-Cell Lung Cancer | N |
| NANDI A, 2019, OXID MED CELL LONGEV | Role of Catalase in Oxidative Stress- and Age-Associated Degenerative Diseases | Y |
| AVCI P, 2013, SEMIN CUTAN MED SURG | Low-level laser (light) therapy (LLLT) in skin: stimulating, healing, restoring | Y |
| EZZEDINE K, 2015, LANCET | Vitiligo | Y |
| BERTRAND A, 2015, BMC MED | Immune related adverse events associated with anti-CTLA-4 antibodies: systematic review and meta-analysis | N |
| GOGAS H, 2006, NEW ENGL J MED | Prognostic Significance of Autoimmunity during Treatment of Melanoma with Interferon | N |
| HUA C, 2016, JAMA DERMATOL | Association of Vitiligo With Tumor Response in Patients With Metastatic Melanoma Treated With Pembrolizumab | N |
| TEULINGS HE, 2015, J CLIN ONCOL | Vitiligo-like depigmentation in patients with stage III-IV melanoma receiving immunotherapy and its association with survival: a systematic review and meta-analysis | N |
| RIBAS A, 2005, J CLIN ONCOL | Antitumor Activity in Melanoma and Anti-Self Responses in a Phase I Trial With the Anti-Cytotoxic T Lymphocyte–Associated Antigen 4 Monoclonal Antibody CP-675,206 | N |
| ALIKHAN A, 2011, J AM ACAD DERMATOL | Vitiligo: a comprehensive overview Part I. Introduction, epidemiology, quality of life, diagnosis, differential diagnosis, associations, histopathology, etiology, and work-up | Y |
| SIBAUD V, 2018, AM J CLIN DERMATOL | Dermatologic Reactions to Immune Checkpoint Inhibitors : Skin Toxicities and Immunotherapy | N |
| BERGQVIST C, 2020, DERMATOLOGY | Vitiligo: A Review | Y |
| PAULOS CM, 2007, J CLIN INVEST | Microbial translocation augments the function of adoptively transferred self/tumor-specific CD8+ T cells via TLR4 signaling | N |
| TAÏEB A, 2007, PIGM CELL RES | The definition and assessment of vitiligo: a consensus report of the Vitiligo European Task Force | Y |
| DAMSKY W, 2017, J AM ACAD DERMATOL | JAK inhibitors in dermatology: The promise of a new drug class | Y |
| SANLORENZO M, 2015, JAMA DERMATOL | Pembrolizumab Cutaneous Adverse Events and Their Association With Disease Progression | N |
| WANG PF, 2017, FRONT PHARMACOL | Immune-Related Adverse Events Associated with Anti-PD-1/PD-L1 Treatment for Malignancies: A Meta-Analysis | N |
| RASHIGHI M, 2014, SCI TRANSL MED | CXCL10 is critical for the progression and maintenance of depigmentation in a mouse model of vitiligo | Y |
| FRISOLI ML, 2020, ANNU REV IMMUNOL | Vitiligo: Mechanisms of Pathogenesis and Treatment | Y |

**Table S3.** Top 50 Affiliations with the Highest Productivity and Specific Parameters

| **Ranking** | **Affiliation** | **Articles** |
| --- | --- | --- |
| 1 | EGYPTIAN KNOWLEDGE BANK (EKB) | 399 |
| 2 | HARVARD UNIVERSITY | 170 |
| 3 | HARVARD UNIVERSITY MEDICAL AFFILIATES | 125 |
| 4 | INSTITUT NATIONAL DE LA SANTE ET DE LA RECHERCHE MEDICALE (INSERM) | 117 |
| 5 | CAIRO UNIVERSITY | 106 |
| 6 | UNIVERSITY OF CALIFORNIA SYSTEM | 92 |
| 7 | CHINESE ACADEMY OF SCIENCES | 89 |
| 8 | UNIVERSITY OF TEXAS SYSTEM | 89 |
| 9 | KAOHSIUNG MEDICAL UNIVERSITY | 88 |
| 10 | UNIVERSITY OF AMSTERDAM | 87 |
| 11 | POST GRADUATE INSTITUTE OF MEDICAL EDUCATION AND RESEARCH (PGIMER), CHANDIGARH | 86 |
| 12 | UNIVERSITE PARIS-EST-CRETEIL-VAL-DE-MARNE (UPEC) | 81 |
| 13 | ASSISTANCE PUBLIQUE HOPITAUX PARIS (APHP) | 80 |
| 14 | CHINESE ACADEMY OF MEDICAL SCIENCES - PEKING UNION MEDICAL COLLEGE | 72 |
| 15 | UNIVERSITE DE BORDEAUX | 72 |
| 16 | HARVARD MEDICAL SCHOOL | 71 |
| 17 | LOYOLA UNIVERSITY CHICAGO | 71 |
| 18 | GHENT UNIVERSITY | 69 |
| 19 | HENRY FORD HEALTH SYSTEM | 69 |
| 20 | TEHRAN UNIVERSITY OF MEDICAL SCIENCES | 67 |
| 21 | CHU BORDEAUX | 66 |
| 22 | GHENT UNIVERSITY HOSPITAL | 63 |
| 23 | YONSEI UNIVERSITY | 62 |
| 24 | IRCCS ISTITUTI FISIOTERAPICI OSPITALIERI (IFO) | 60 |
| 25 | CATHOLIC UNIVERSITY OF KOREA | 58 |
| 26 | IRCCS SAN GALLICANO DERMATOLOGICAL INSTITUTE (ISG) | 58 |
| 27 | **UNIVERSITY OF MASSACHUSETTS SYSTEM** | 58 |
| 28 | AIR FORCE MEDICAL UNIVERSITY | 57 |
| 29 | **UNIVERSITY OF MASSACHUSETTS WORCESTER** | 56 |
| 30 | ALL INDIA INSTITUTE OF MEDICAL SCIENCES (AIIMS) NEW DELHI | 55 |
| 31 | NORTHWESTERN UNIVERSITY | 55 |
| 32 | FUDAN UNIVERSITY | 54 |
| 33 | HENRY FORD HOSPITAL | 54 |
| 34 | MASSACHUSETTS GENERAL HOSPITAL | 54 |
| 35 | UNICANCER | 53 |
| 36 | UNIVERSITE PARIS CITE | 53 |
| 37 | KING SAUD UNIVERSITY | 52 |
| 38 | YONSEI UNIVERSITY HEALTH SYSTEM | 52 |
| 39 | ACADEMIC MEDICAL CENTER AMSTERDAM | 51 |
| 40 | CENTRAL SOUTH UNIVERSITY | 51 |
| 41 | NATIONAL TAIWAN UNIVERSITY | 51 |
| 42 | UNIVERSITY OF NOTTINGHAM | 51 |
| 43 | MEMORIAL SLOAN KETTERING CANCER CENTER | 50 |
| 44 | ZHEJIANG CHINESE MEDICAL UNIVERSITY | 48 |
| 45 | CHANG GUNG MEMORIAL HOSPITAL | 47 |
| 46 | JOHNS HOPKINS UNIVERSITY | 47 |
| 47 | PEKING UNION MEDICAL COLLEGE | 47 |
| 48 | HOPITAL UNIVERSITAIRE HENRI-MONDOR - APHP | 45 |
| 49 | TEL AVIV UNIVERSITY | 44 |
| 50 | UNIVERSITY OF TEXAS SOUTHWESTERN MEDICAL CENTER | 44 |
